# Developing a contracts law keyword list (CLKL) for academic legal education: A corpus-based, keyness-informed study

**DOI:** 10.1371/journal.pone.0352766

**Published:** 2026-07-06

**Authors:** Abdullah Alasmary

**Affiliations:** Department of English Language and Translation, College of Language Sciences, King Saud University, Riyadh, Saudi Arabia; UKM: Universiti Kebangsaan Malaysia, MALAYSIA

## Abstract

This study introduces the Contracts Law Keyword List (CLKL), a discipline-specific, genre-focused resource of pedagogically useful lexical items in contract law, an understudied legal subfield at the nexus of law and commerce. Items in the CLKL are drawn from authentic language data using predetermined frequency, range, and keyness parameters. The CLKL includes 747 keywords (KWs) typical of written contract law textbooks, covering 4.22% of all words in the study corpus and 4.19% in the law section of the British National Corpus (BNC). Structural analysis revealed the pervasive presence of nouns, which account for nearly half of all grammatical categories. Adjectives and verbs constitute the second- and third-largest groups of KWs. There are rare instances of adverbs, archaic terms, prepositions, and words with dual structural functions. These findings underscore the importance of creating a field-specific vocabulary list that can address the lexical demands of a growing number of law students and international practitioners. The CLKL may also be used to deepen knowledge of domain-specific vocabulary, enhance engagement with authentic language examples, and foster awareness of expert-authorized conventions typical of legal contracts. Law educators can draw on the list while designing instructional materials or planning classroom activities.

## Introduction

In recent years, the number of international students pursuing academic law degrees at English-medium institutions has increased substantially [1,2]. Legal education is a thriving global enterprise, and programs such as the Legum Magister (LL.M.) and Doctor of Juridical Science (S.J.D) attract a rapidly growing number of students and practitioners from different language backgrounds [2]. Early-career lawyers and novice law academics who receive professional education in their native languages may decide to pursue short-term training programs where English is the medium of instruction [1]. Although some foreign-trained students and international legal practitioners demonstrate higher levels of English proficiency, they still encounter substantial challenges in a domain as multilayered as law. Several researchers highlight that the need for classroom-based interventions to challenge the complex nature of the legal language [3–6]. At the lexical level, legal texts are replete with polysemous and synonymous words, culture-specific expressions, binominal phrases, archaic borrowings, and abbreviations [5]. Other important features characterizing the legal lexicon include the use of “doublets, polysyllabic words, compound and unusual prepositional phrases, and passive verbs” [3, p. 311]. The third layer of difficulty stems from the ubiquitous presence of familiar words with law-specific meanings. For example, modal verbs, such as shall and must, are key legal instruments used to “impose a high degree of obligation on the addressee” [6, p. 34]. Syntactically, legal sentences are relatively long and structurally complex, with a marked preference for nominal elements, which are required for precision and clarity [4].

While some previous studies have compiled similar word lists pertaining to contract law [7,8], the list reported in this study is unique in three important ways. First, it draws on textbooks as its source genre to generate a pedagogically relevant and linguistically meaningful list tailored to meet the disciplinary and lexical demands of novice law learners and early-career practitioners. The list created by Hanks et al. [7], by contrast, is derived from a corpus of actual contracts and aims to fulfill the needs of business professionals, rather than law students. Second, this study offers a more comprehensive list of keywords than the list compiled by Alasmary [8] who selected the top 100 items only. These items are pedagogically useful, but lack extensive coverage which is a key parameter in vocabulary studies, revealing the “extent to which words in a text are known” to the target discourse community [9]. A third important contribution of this study lies in the adoption of a keyness approach as a methodological framework for identifying and selecting vocabulary words of pedagogical value. The notion of keyness is used in corpus linguistics to refer to a lexical item that “occurs with unusual frequency in a given context … by comparison with a reference corpus” [10, p. 236]. To compute keyness, this study pursues the step-by-step approach developed by Gabrielatos [11], who advocated the use of effect size measurements for locating and extracting lexical items, ensuring that the identified items are not only statistically significant but linguistically meaningful. Third, the list reported in this study is accompanied by research-informed, evidence-based description of several classroom intervention approaches aimed for optimizing the use of list items.

### Law learners’ needs

It is estimated that approximately one million international students are enrolled in various academic institutions across the United States [12]. There are several challenges facing these students, the most significant of which is limited knowledge of the English language. Oduwaye et al. [13] surveyed the problems that international students encounter while pursuing their studies in English-speaking countries and concluded that “language use might be the underlying cause of all major issues international students encounter” [13, p. 9]. Applied linguistics researchers seem to agree that the multilayered nature of law language requires aligning instructional intervention and pedagogical practices with the needs of the students [14,15]. This is particularly evident in the longitudinal study carried out by Xu and Casal [15], who demonstrated that extended focus on lexical sophistication and genre-specific conventions can significantly shape the developmental trajectories of legal writers. A thorough understanding of and meaningful engagement with legal discourse over an extended period of time supports the evolving needs of L2 legal learners who are required to interact with members of the legal community through a variety of channels, including writing. In much the same vein, Hartig [14,16] draws attention to the importance of disciplinary concepts in shaping students’ ability to read and write legal texts effectively. In this case, learners need support not only in mastering legal terminology, but also in navigating the conceptual underpinnings of legal discourse, such as the use of precedent and analogical reasoning. A distinction, therefore, needs to be made between two interrelated concepts: discourse-structuring and discourse-relevant vocabulary,

### Specialist vocabulary listings

Over the past few decades, researchers have attempted to produce several vocabulary lists to help learners navigate the complexities of academic and non-academic disciplines [17–20]. West [17] made the first attempt to develop a list of potentially useful vocabulary that can be used for pedagogical purposes. He created the General Service List (GSL), including nearly 2,000 word families. Items on the list were selected based on several criteria, such as frequency of occurrence, ease of use, and text coverage. Despite its huge impact and widespread use, the list remains outdated and was heavily influenced by the compiler’s subjective judgment [17]. Focusing on academic English, Coxhead [18] built a three-million-word corpus of journals and academic textbooks representing four disciplines: the arts, commerce, law, and science. The final list, termed AWL, comprises 570-word families, which accounted for 10% of the tokens in the entire study corpus. The list is so influential that it has informed much of the research and practice in English for Specific/Academic Purposes. Similarly, Brezina and Gablasova [19] introduced the New GSL, consisting of 2,494 lemmas derived from four corpora. The criteria for selecting words in the list include the frequency of occurrence, dispersion across different corpus subparts, and the extent to which an item occurs across these different corpora. Although the list distills data in a way that addresses shortcomings of earlier list-building efforts, some items offer little pedagogical value, as their meanings are transparent (e.g., Internet, website, and e-mail). Cobb and Laufer [20] produced the Nuclear Family List comprising 2,887 word families from two general corpora: the Corpus of Contemporary American English (COCA) and the British National Corpus (BNC). The list is intended to serve the needs of learners opting to master general English. Although most lists are derived from written corpora, few others are based on the spoken language. For example, Dang [21] created the Academic Spoken Word List (ASWL), designed to help learners understand the language of academic conferences. Given its wide coverage, this list is expected to facilitate the delivery of research papers or conference participation.

In an apparent reaction to criticism leveled against general vocabulary lists, several researchers have produced lists of target vocabulary typical of specific disciplines, registers, and genres [22–25]. Tongpoon-Patanasorn [24] compiled a two-million-word corpus from which a list of technical KWs in the finance domain was selected. Candidate KWs were then provided to three disciplinary experts, and non-technical words were excluded. The final list comprised 979-word families headed by 569 words. At the top of the list are words such as financial, value, debt, firm, and cash, all of which appear to be related to finance. In a similar study, Watson-Todd [25] used a keyness approach to produce a list of KWs derived from a 1.3-million-word corpus of engineering textbooks. After applying several filtering procedures, the analysis yielded 186 words, of which 40 were identified as opaque terms that, as found after examining concordance lines, had technical meanings different from their common use (e.g., constant, column). Another list was developed by Lei and Liu [22] to select words that are characteristic of the medical domain. The list consisted of 819 lemmas to present medical students with a resource of field-specific vocabulary drawing on two corpora of journal articles and academic texts. Focusing on nursing, Yang [26] compiled a 676-word list from a corpus of informational booklets and compared the selected items with other academic word lists. In a study focusing on agriculture, Martínez et al. [23] elicited academic vocabulary from a corpus of journal articles. They divided words into subgroups according to their occurrence in article sections: Introduction and Methods. Academic words emerging from the corpus analysis were compared against items on GSL and AWL, and some words, although previously highlighted as having an academic meaning, mirrored technical usage in agriculture (culture). The vocabulary list for pre-university students, suggested by Green and Lambert [27], was derived from a corpus of textbooks on eight disciplines that can be broadly grouped into two major categories: hard sciences (e.g., biology) and humanities (e.g., geography). After applying a set of criteria, such as frequency, dispersion, and range, the final list included 4,781 lemmatized words, with biology having the largest number of words and mathematics the lowest. Coxhead et al. [28] suggested a recently compiled list derived from 1,079-word types from a corpus of spoken and written materials on fabrication, a subfield of mechanical engineering. Within the legal domain, Bancroft-Billings [29] recorded classroom interactions in a contract law course and compiled a list of key technical legal vocabulary by carefully analyzing transcripts. Although it draws on a small corpus, the final list is pedagogically useful because it comprises domain-specific terms and expressions. Combining the items on Bancroft-Billings’ [29] list with those emerging from this study, students and potential users can find both sources useful for addressing the oral and written aspects of vocabulary in contract law.

Producing vocabulary lists using different approaches has recently blossomed owing to growing awareness among researchers regarding the need to help students gain a deeper understanding of, and appreciation for, the language patterns emerging from actual language data [30,31]. However, these compiled lists are tied to a specific discipline (engineering) or genre (journal articles and textbooks). To extend this line of inquiry, other understudied disciplines and overlooked genres need to be explored for key vocabulary patterns shaping these disciplines and genres. Thus, this study is unique in its focus on contract law as a legal subdiscipline and academic textbooks as an essential part of a student’s educational life.

### Legal English

For a long time, the language of law (legalese) has been the focus of increasing scholarly activity. Such a language is sometimes described as “overly complicated, dense, repetitive, and outdated” [3, p. 303]. Legal language is also seen as “decidedly peculiar and often hard to understand, especially from the perspective of the lay public” [32, p. 2]. The nature of legal vocabulary, which is replete with archaic and rarely used words, nominal constructions, and Latinized expressions, is another difficulty [33]. Townley and Jones [34] point out that many terms typical of legal writing “have unrelated everyday meanings, and the language is often arcane and abstruse” [34, p. 71]. Legal English has received considerable attention in linguistics and in English for specific purposes. In a study that investigated the interplay between a general language course and a domain-specific one, Baffy [1] reported several language-related challenges facing non-English-speaking students pursuing their studies at US-based institutions. As an intervention to address these challenges, Baffy suggests that English for Legal Purposes programs “should offer courses that draw on subject-specific content that helps to develop students’ fluency for academic tasks” [1, p. 68]. Hartig and Lu [2] examined passive and nominalized forms in judiciary memos. They found that these patterns occurred more frequently in texts produced by experts than in texts written by novices, a conclusion that warrants a pedagogical intervention to help students bridge this expert-novice gap. Focusing on e-mails and cover letters of a legal nature, Townley and Jones [34] showed that the negotiation process involved in producing these genres employs a combination of strictly legal language and ordinary expressions to finalize a contractual agreement. Using a corpus-based approach, Vass [35] compared the use of hedging verbs in majority and dissenting court opinions and law journal articles, showing that the use of specific hedging verbs is inextricably linked to and shaped by the communicative purpose of each setting. Determining a methodologically appropriate approach for extracting legal terms from a corpus of UK Supreme Court decisions was the purpose of the study conducted by Pérez and Rizzo [36], who showed that using computerized programs in defining, locating, and extracting specialized vocabulary from language data has far-reaching implications such as creating legal glossaries, fostering proper translation, and developing data-driven materials for classroom instruction. In a survey of several written legal materials, Candlin et al. [3] concluded that most law textbooks are heavily biased in favor of legal content rather than the language of the law.

This study aims to derive, analyze, and present keywords typical of the academic study of contract law. It fills a prominent gap in the literature, as no previous attempt has been made to create a domain-focused, genre-based list of key vocabulary in contract law that can be used by beginner lawyers, novice law students, or foreign legal practitioners. The vocabulary items on the list may be pedagogically useful in informing current teaching practices and helping to create instructional materials for legal purposes in English. In order to guide the process of making this list, answers to the following questions are sought:

1. Which lexical items appear as keywords in a corpus of written academic textbooks on the law of contracts?
2. What teaching approaches can be pursued for instructional intervention in an English for Legal Purposes (ELP) program?

## Materials and methods

### Study corpus (SC)

This study draws on a 3-million-word corpus of 20 full-length academic texts (S1 Appendix) topically and thematically linked to the study of contract law. According to Kurzon [37], academic texts are an important sub-area of legal language. The impetus for deriving KWs from textbooks rather than from any other academic genre is motivated by three factors. First, textbooks are a dominant academic genre, and their presence in students’ lives is unavoidable. Second, textbooks are fundamental for knowledge creation and dissemination as they remain “one of the primary means by which the concepts and analytical methods of a discipline are acquired” [37, p. 112]. Law textbooks occupy a prominent status as they are “the most commonly used genre of the written language of the law in pedagogic settings” [38, p. 229]. A third factor motivating this study is the scarcity of data-driven learning materials, broadly conceptualized as resources developed from large collections of authentic written or spoken texts. These resources are customized to address the needs of international students willing to pursue further academic degrees or seek opportunities to practice law in contexts where English is the medium of legal instruction. Given the field-specific and genre-focused nature of this study, only written academic texts in contract law were used to build the SC from which the target KWs were derived.

S1 Appendix includes all the textbooks used to build the study corpus. The process of selecting these textbooks was informed by four key considerations: topic-specificity, publication date, authorship, and their communicative purpose. All texts have titles indicative of their topical focus on contract law. Eleven texts were single-authored, and four were co-authored. Three writers authored one book, and multiple contributors edited four volumes. Although the texts vary regarding their subtopics, they share some commonalities such as the focus on the foundational principles underlying the law of contracts (e.g., offer, promise, and acceptance), contractual relations (e.g., mistake, breach of a contract, discharge, and remedies), and rights and obligations. Only a few textbooks examine the epistemological and historical foundations of the law of contracts. All texts were produced by key publishers known for their academically oriented books with worldwide reputations and readership. The selected texts were published between 2001 and 2020 and are accessible to the author through an institution’s electronic database. Before the corpus analysis, all texts were thoroughly refined, thus removing reference lists, page numbers, headers and footers, footnotes and endnotes, tables of cases, legislation and statutes, and indices with the aim of focusing only on the content. In summary, the choice of texts in the SC took into consideration the topical focus of each text (contract law), time of publication (2000–2020), the communicative purpose of the data source (written academic textbooks), and authorship (academics and law experts).

### Reference corpus (RC)

To compute keywords, a reference corpus (RC) was built from fifty-five texts representing many legal topics, none of which were related to the law of contracts. Examples of topics addressed include child, energy, aviation security, animal, and financial laws. The RC is three times larger than the SC in terms of both overall texts and running words. Table 1 provides a brief description of the study and reference corpora.

**Table 1:** Composition of study corpus and reference corpus.

Corpora	# Texts	# Running words	# Types
Reference corpus (RC)	55	9,290,475	17,963
Study corpus (SC)	20	3,383,041	20,341

### Keywords seclection and filtering

KWs were determined using various methodological interventions, the most common of which are frequency-based comparisons of a predefined set of linguistic patterns in two corpora: SC and RC. The study corpus comprises texts on the law of contracts aimed primarily for students of law. The reference corpus, in contrast, covers broader law-related topics and is constructed for comparison purposes. Statistical measures such as log-likelihood and chi-square tests were applied to establish the statistical significance of observed frequency differences. Although this approach is still widely used, it has been criticized for inadequately capturing the full extent of frequency differences and, therefore, failing to measure keyness. Gabrielatos [11, p. 231] noted that “statistical significance is not an appropriate metric for keyness; rather, keyness needs to be established via an effect-size metric.” A corpus-generated list whose items are arranged using an effect-size approach is expected to help language-teaching instructors and course designers prioritize items that address students’ lexical demands, given their keyness. There are multiple methods for calculating effect sizes, each of which has its advantages and limitations. This study adopts Everitt’s [39] formula as it emphasizes raw frequencies—the actual occurrences of a lexical item obtained through corpus analysis—over normalized frequencies, which involves adjusting raw frequency counts relative to the corpus size, a procedure needed when comparing lexical items across corpora of different sizes.

After preparing documents for corpus analysis and as a first step, all candidate KWs were obtained using the keyword function in WordSmith Tools 8 [40]. The minimum word frequency was set to five times per million words (p.m.w.), a relatively low cutoff score that allowed for a greater number of candidate KWs to be included in the initial list. Another important consideration at this stage concerns the distribution of the target candidate KWs in the corpus subparts. Although there is no consensus among researchers on a cutoff distribution score beyond which a certain word is selected for analysis, scholars are increasingly aware of the importance of such criteria in corpus studies [41]. The distribution score was set at 25% for all the texts constituting the entire corpus. This relatively high distribution score ensures that the target keyword does not reflect the idiosyncratic use of a particular author, nor does it feature a specific book style or subtopic. WordSmith tools are used to compute Log-likelihood (G2) and Bayesian Information Criterion (BIC), used to establish the statistical significance of the frequency differences, and to give a preliminary keyness-rated list. Notably, very few subgroups of candidate KWs occur in the SC but with zero frequency in the RC. Applying an effect-size procedure was not applied because of the zero scores. Gabrielatos [11] suggests that an item with a zero frequency in the RC is replaced with an “infinitesimally small number (0.000000000000000001),” which indicates instances of absences.

Applying the above-mentioned selection criteria returned 2,021 candidate KWs; all had a BIC score greater than 10 (BIC > 10), the threshold indicating very strong evidence against the null hypothesis. All candidate KWs were copied and entered an Excel spreadsheet to compute effect sizes using Everitt’s [39] formula. The list from the application of the effect size requires further scrutiny to eliminate items that are less pedagogically appealing. First, several words (576 words) are either proper or place names (Hadley, London) that need to be eliminated from the list. Second, keyword analysis returned 48 acronyms and abbreviations. By examining these concordances, it becomes clear that some abbreviations and acronyms may denote more than one notion. For example, the acronym MLR is used to refer to medium lending rates in some contexts but stands for Money Laundering Regulations in others. Although some are important, all acronyms and abbreviations were removed from the preliminary list because of their dual referents. The third procedure involved removing 30 words whose meanings posed no obvious challenge, given their transparent and salient uses. Examples include pronouns (you, he), nouns frequently used in everyday conversation (uncle, nephew, e-mails), and titles (Mr., Sir.). In the fourth step, the candidate KWs in the GSL and the AWL were deleted from the list. The impetus for this procedure is twofold. First, law students and novice professionals for whom this list was created “are advanced speakers of English who should be familiar with words from the GSL and a significant portion of the AWL” [29, p. 13]. The second reason is to avoid overburdening potential users with an inflated list that, given class-time constraints, may not be entirely covered [42].

In order to facilitate its inclusion in mainstream English for Legal Purposes programs, all KWs in the list were tagged for part of speech (POS-tagging) using CLAWS, a program with an error rate of less than 6% [27]. Each POS-tagged item was manually checked to ensure that it was assigned to the correct grammatical class (e.g., noun, verb, adjective, etc.). Part of the criticism leveled against Coxhead’s [18] list is the lack of information about the part of speech to which a vocabulary item belongs. Therefore, the current study addresses this shortcoming by providing POS tagging.

Finally, it is essential to point out that the procedures implemented to derive and distill data are intended to ensure the pedagogical relevance of items on the list for law education and substantiate that the list mirrors the language content that the intended audience, students and early-career practitioners, may encounter in their studies and professional life.

## Results and discussion

**Fig 1:**
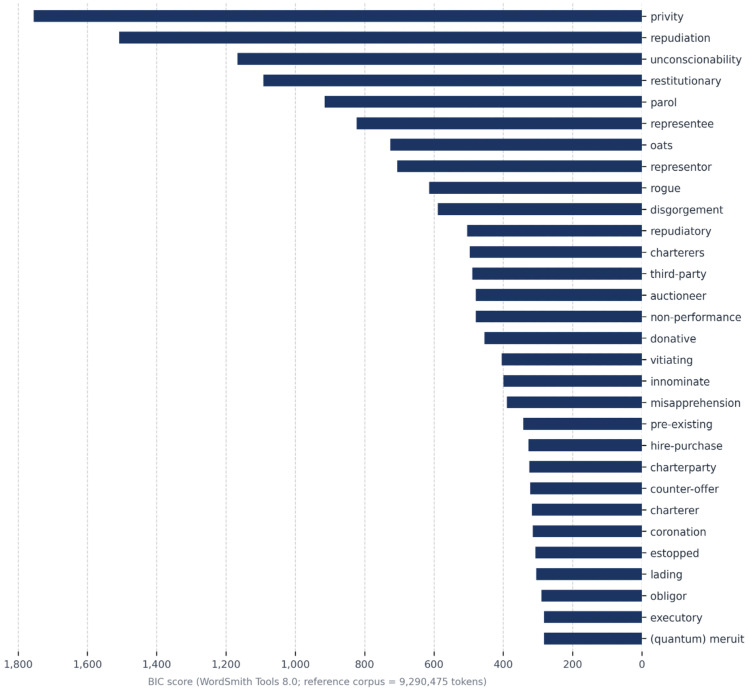
Top 30 keyness-ranked keywords.

Corpus analysis and subsequent filtering procedures produced a list of 747 items arranged in descending order based on their keyness scores (S2 Appendix). Fig 1 demonstrates the top 30 items resulting from keyness measures. Privity, repudiation, unconscionability, restitutionary, and parol were ranked as the top five key items on the list, whereas donee, cancellation, defects, construed, and evidentiary were the least key items. The list items are not sorted into distinct word families [18], nor are they arranged into lemmas, that is, word members belonging to the same lexical class [43]. The keyness analysis is therefore the guiding principle in ranking patterns in the list. In this case, repudiation is more key than lemmatized forms such as repudiatory (ranked 11), repudiating (ranked 72), repudiates (ranked 123), and repudiate (ranked 427).

**Fig 2:**
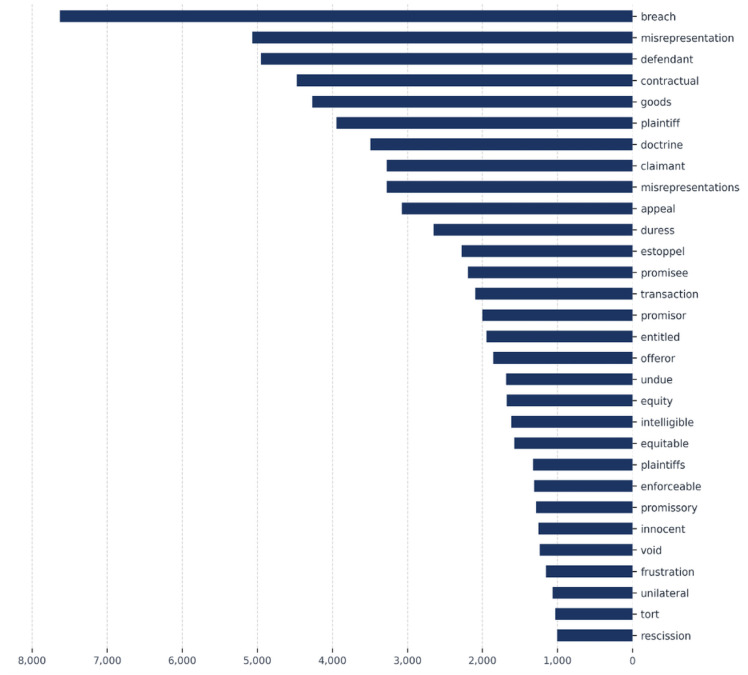
Top 30 frequency-ranked keywords.

As can be seen in Fig 2, a frequency-based ordering of items yielded a somewhat different ranking, with items such as *breach, misrepresentation,* and *defendant* prioritized over more domain-specific items of *privity, repudiation,* and *unconscionability.* While sorting by frequency is a plausible approach, Keyness ordering of items better captures the “aboutness” of a specialized corpus.

The grammatical analysis of items on the CLKL revealed some interesting tendencies. As Table 2 illustrates, the CLKL is dominated by 370 key nouns, representing nearly 49% of all items on the list. The ubiquitous presence of nouns in the list is not surprising, given that nouns are a key feature of more sophisticated writing [44]. Nouns are observed to “create an impression of greater objectivity” and are therefore “considered to be especially important in cases of fact-stating” [5, p. 32]. Adjectives occupy the second largest grammar pattern, totaling nearly a quarter of all KWs in the list. Notably, adjectives are more important in the CLKL than verbs, a finding that runs counter to what has been established in previous corpus-based lists [19,43]. Mattila [5] explained that the ubiquitous use of adjectives in legal discourse is due to their dual grammatical functionality (e.g., the accused and the insured). Verbs make up a relatively small proportion (14%), whereas adverbs and archaic terms comprise the smallest groups, comprising 24 and 20 KWs, respectively. The prevalent use of nouns and the considerably limited presence of other grammatical categories do not imply that these constructions are of little importance. The distribution of these patterns in a corpus is influenced by several factors, the most important of which are the type of register under investigation, the inclusion/exclusion parameters, frequency of occurrence, and dispersion across the corpus sub-parts. Given that some of these archaic words are part of a multi-word string with a tendency to co-occur in most contexts (prima facie and per annum), the key archaic pattern is given as a single multi-word expression. A small number of KWs in the list serve a dual function tied to a specific context. The grammatical category of patterns such as common sense and repudiating is only determined by a close examination of the concordance lines as they behave differently in different contexts.

**Table 2:** Structural categories of the 747 keywords identified in the study corpus.

Word Class	No.	%	Example
Noun	370	49.6	privity, repudiation, unconscionability
Adjective	186	24.9	restitutionary, donative
Verb	112	14.9	estopped, vitiated
Adverb	24	3.3	innocently, unconscionably
Archaic	20	2.7	(quantum) meruit, (contra) proferentem
Pronoun	1	0.1	whosoever
Preposition	1	0.1	vis-à-vis
Context-dependent	33	4.4	commonsense, repudiating

With respect to coverage, the results show that the CLKL covers 4.22% of tokens in the entire SC. Other studies have reported far greater proportions: 23.2% [43], 20.18% [22], and 15.43% [45]. The primary reason for this comparatively lower coverage lies in the different keyness analyses pursued in this study and the discipline-specific nature of the SC. Another reason is the exclusion of items from the GSL and AWL lists, which, if included, may increase coverage but would inflate the list. Nevertheless, legal students and early-career law practitioners are expected to find the CLKL a pedagogically useful source for improving text comprehension and intelligibility.

Comparing the CLKL with existing lists and specialist corpora revealed some interesting findings. Table 3 shows that the CLKL accounts for 4.19% and 3.11% of running words in the law sections of the BNC and British Academic Written Corpus (BAWE), respectively. The relatively high coverage of the CLKL in the law section of the BNC attests to the viability and representativeness of items in the CLKL. Although contract law and social sciences are not entirely distant fields, the relatively low coverage of the CLKL (2.92%) in the corpora of these disciplines demonstrates the field-specific focus of items. Notably, the academic nature of the texts comprising the CLKL explains the comparatively higher coverage (3.11%) of the CLKL in BAWE. Interestingly, the CLKL covers of only 3.09% of the commerce sub-corpus of the BNC, although contract law and commerce are two closely related fields.

**Table 3:** Coverage of the CLKL across four comparative corpora.

	BAWE	BNC (Law)	BNC (Social Science)	BNC (Commerce)
Coverage (%)	3.11	4.19	2.92	3.09
Corpus size (million words)	1	2.2	2.3	2.2

### Domain-specific vs. overlapping keywords

The lexical analysis for wordlist construction often reveals concepts unique to the domain under investigation, as well as terms that occur in other genres and disciplines. Domain-specific vocabulary items are inextricably linked to the discourse markers, conventions, and knowledge structures within the field of study, whereas overlapping patterns also occur in other areas and may serve different contextual meanings. One way to establish the domain-specific nature of the CLKL is through a comparison with available lists representing different disciplines. A full-scale comparison is not feasible, given the various criteria by which each list was created. The agricultural list compiled by Martínez et al. [23] contains two items found in the CLKL: induced and established. The medical list suggested by Lei and Liu [22] shares 17 word types with the CLKL. Examples include performance, case, binding, and compliance. The same shared word may express layers of different meanings depending on the domain and context in which it occurs. For example, the word oral is associated with a technical procedure in medicine, whereas contract law uses this term to denote the way a contract may come into existence. The nursing list of academic vocabulary contained 11 words that also appeared in the CLKL. Items such as well-being, compliance, and discharge are examples of shared language. Notably, these words behave differently according to the discipline. The word discharge refers to the termination of a contract without holding either party legally responsible. In medical discourse, discharge carries different meanings; the most common is the act of releasing a patient from care.

Clearly, several identified KWs have a meaning closely tied to the law of contracts. However, some expressions may be used for general communication and, though they can still carry a discipline-specific meaning. The word *party* can be used as a standalone expression, but it can also be employed to create hyphenated compounds, such as third-party, charter-party, and three-party. The following examples drawn from the corpus show context-dependent usage:

1. *“The House of Lords held that a clause in a charter party of a ferry did not cover the financial loss suffered by charterers.*”
2. *“Can Prime sue Prime as a third-party beneficiary of the contract between Prime and Owners?”*
3. *“It is rare to find an excessive bargain jurisdiction pleaded in a three-party situation; however, in theory, the same notice principles should apply equally to it.”*

Another important tendency in the list is the use of concepts that lean heavily to the practice of the contracts law. KWs such as *acceptor*, *obligee*, and *payor* are deeply rooted in the language of legal contracts but are rarely used for general communication:

1. *“It would be better if, as soon as the letters were posted, the acceptor could proceed on the basis that a contract had been made.”*
2. *“As permitted by its constitutive documents, the obligee was voluntarily liquidated.”*
3. *“Under this regime, a mistaken payor fully internalizes the costs of the wrong payment.”*

### Keywords in thematic groups

To gain a deeper understanding of the list and to facilitate its subsequent pedagogical use, a total of 13 thematic groups were identified to account for clusters of semantically similar keywords. The thematic groups appear to mirror the key successive phases of a contract: formation, interpretation, breach and remedy. Table 4 lists these groups along the total number of keywords in each group. As can be seen, the greatest number of keywords are used either to highlight parties and actors involved in establishing contractual relations or to create specific contractual conditions for such relations to exist. Four other groups of semantically congruent keywords are used for creating evaluative and descriptive conditions, identifying vitiating factors, formulating contractual terms, and remedying breaches. Other distinct groups, though less frequent than the previous ones, include keywords that revolve around concepts of contract formation, breach and performance, and contract formation and interpretation. At the far end of the semantic categorization scheme are vocabulary key items on legal concepts and procedures, and archaic and Latin-derived keywords which reflect the historical background of modern legal reasoning and thinking.

**Table 4:** Keywords in distinct semantic groups.

Keywords per Thematic Group (N = 747)		
Group	Category	Keywords
G1	Parties and Actors	93
G2	Contract Formation	52
G3	Vitiating Factors	70
G4	Contractual Terms	66
G5	Breach and Performance	54
G6	Discharge and Termination	37
G7	Remedies	64
G8	Equity and Legal Doctrines	24
G9	Interpretation and Construction	59
G10	Procedural and Judicial Language	38
G11	Archaic and Latin Expressions	21
G12	Specific Contractual Contexts	92
G13	Evaluative and Descriptive	77

Within each group, keywords may cluster around even narrower semantic meanings. For example, the first sematic group comprises keywords on contractual parties and agents. Some of these keywords account for core contractual parties (e.g., *offeror/offeree, promisor/promisee, obligor/obligee, acceptor, payee/payor*), while others assign litigation roles to individuals (e.g., *claimant, defendant, plaintiff, appellant, representee/representor*).

1. “We must understand why we require an *offeree* to take steps to bring an acceptance to the *offeror’s* attention at all.”
2. “It is not clear that a *claimant* should lose his right to terminate simply because he was unaware of his right to do so when he offered a bad reason for terminating.”

The second largest semantic group of keywords may also be divided into broad sub-units, namely maritime and carriage contracts (e.g., *charterparty, lading, freight)*, property and conveyancing (e.g., *tenancy, mortgage, lease),* construction and industry (e.g., *carpentry, collieries, demolition*), and commercial and financial transactions (e.g., *auctions, invoice, mercantile*).

1. “Hire rate to be ‘equitably’ adjusted under a *charterparty.*”
2. “The issue before the court was whether a term could be implied into the *tenancy* agreement.”

Keywords performing descriptive and evaluative functions can also be grouped under smaller categories. For example, several keywords are used to reflect a level of emphasis (e.g., *manifestly, exceptionally, utmost*). Others may also be functionally employed to invoke moral and ethical condemnation (e.g., *reprehensible, oppressive, fraudulently, inequitable, harsh*), maintain fairness (e.g., *reasonableness, unreasonable, unfairness, aggrieved, amity*), and identify party conditions (e.g., *innocent, weaker, equivocal, wary, indifferent*). Another subgroup involves lexical items that are used to account for misrepresentation and deception (e.g., *misrepresentation, deceit, falsity*), duress and undue influence (e.g., *duress, coerced, blackmail*), fraud and dishonest (e.g., *fraud, wrongdoing, opportunism*), and mistake and incapacity (e.g., *intoxication, cross-purpose, misapprehension*). Concepts such as non-reliance, *extortionate*, *improvident*, and *imbalance* belong to a sub-class of keywords demonstrating case description and legal procedures.

1. “It also protects the parties against a choice that is *manifestly* unfair.”
2. “It can be rejected without impairing the sense or *reasonableness* of the contract as a whole.”

The analysis also reveals three groups on themes related to contract law terminology, remedies, and interpretation and construction. Examples include keywords such as *stipulation*, *apportionment*, and *axioms*.

1. According to Article, [a]person may make a stipulation in a contract for the benefit of a third person.
2. “However, there are some cases in which *apportionment* should be awarded.”
3. “The assumption that the disgorgement interest is not protected by contract law rests among the rubble of those *axioms*.”

The remaining set of keywords are thematically tied to concepts of breach and performance, contract formation, procedural and judicial dealings, discharge and termination, and equity and legal doctrines. The smallest thematic group in the list contains expressions of Latin and Greek origin. Each group comprises a closed set of keywords representative of a narrower conceptual domain. For example, keywords on breach and performance can be used to discuss breach and its forms (e.g., breach, defects), performance and non-performance (e.g., counter-performance, dishonoured), and payment and financial obligations (e.g., accrued, arrears). In a similar vein, key items on contract formation are classified into smaller thematic groups such as offer and invitation (e.g., advert, bit), consideration (e.g., affection, bargained-for), and acceptance (e.g., assent, uttered). Litigation process, pleading and claims, and proof of evidence are all sub-groups within the larger thematic category up of procedural and judicial language. Language of discharge and termination is represented in several key groups, including frustration (e.g., frustrate, impracticality), rescission (e.g., rescind, retraction), and termination (e.g., cancel, withdraw). Keywords on equity and legal doctrine may also be subdivided into narrower categories such as estoppel (e.g., acquiesced, estopped) and privity (e.g., mutuality, novation). Here are examples of several keywords representing these groups:

1. “In short, the normal remedy for *breach* of contract is that provided in the contract itself.”
2. “It is suggested that gratuitousness is best judged by whether or not the party undertaking the obligation can or cannot compel a *counter*-*performance* at the time he undertakes the obligation.”
3. “Terms that identify rights and liabilities that have *accrued* prior to frustration continue to operate.”
4. “As to the wide distribution of the *advert*, the court did not regard this as a problem.”
5. “Severe economic crises will not normally *frustrate* the contract.”
6. “A and B will retain the power to vary or ‘*rescind’* the contract without T’s consent.”

The dominant presence of terms accounting for parties and actors, on the one hand, and terms designating specific contractual contexts, on the other hand, reveals the relational and situational nature of the law of contracts. For textbook authors, these two semantically interdependent categories reflect a common objective, that is, navigating the complex web of situations where rights and obligations are constituted. A cluster of six other semantic groups—vitiating factors, contractual terms, remedies, interpretation and construction, breach and performance, and contract formation—shows that contractual relationships are relationally anchored, procedurally elaborated, and doctrinally specialized. The relatively high proportion of evaluative and descriptive terms is unsurprising, given their role across all phases and stages involved in the process of contract formation, interpretation, and implementation. Accounting for 16% of all items, the remaining semantic groups—procedural and judicial language, discharge and termination, equity and legal doctrines, and archaic and Latin expressions—are less proportionally represented. The comparatively lower proportion of such items may be due to several factors such as the substantial overlap with other more proportionally elaborate categories (e.g., procedural and judicial language), the inherently closed nature of the lexical inventory (e.g., archaic and Latin terms), and the distinct situational characteristics of academic textbooks as channels through which subject matter knowledge is constructed and disseminated. As such, textbooks on contracts law address issues related to the conceptual exposition of substantive doctrine rather than the operational tasks of pleading, drafting, and adjudication.

The final, and by far the smallest, thematic category includes keywords of Graeco-Latin origin. Legal language is characterized by several unfamiliar expressions inextricably tied to legal thinking and reasoning. The keyness analysis produced 20 archaic expressions of Latin and Greek origins. Such patterns are unsurprising, given some previous descriptions of legal language as having several “Latinisms” [3] and “Latin borrowings” [5]. Examples of such Graeco-Latin words include *pari delicto*, *culpa in contrahendo*, and *inter praesentes*, all of which appear to have specialized meanings. A term such as *in pari delicto* is used to express equal fault pertaining to contracts, whereas culpa, in contrast, Hendo indicates a fault in the conclusion of a contract; parties entering into a legally binding contract agreement must act in good faith during pre-contractual stages. The phrase *inter praesentes* is used to allude to a legal condition in which contractual parties should meet face-to-face to finalize a deal. These Graeco-Latin words are not pervasive in classroom input; this necessitates direct instructional intervention to explain what they mean and how they are used in academic and professional writing. The list also has two unfamiliar patterns: a preposition (vis-à-vis) and a pronoun (whosoever), which are highly infrequent and somewhat elusive in meaning. Vis-à-vis is a preposition but may also serve as an adjective in some contexts, warranting careful instructional intervention. Whosoever is a pronoun that is commonly reduced to whoever, but in legal texts, it is preferable to retain the archaic term for precision. The examples below provide the context for words such as *meruit, proferentem* and *delicto*:

1. “The developer was awarded a quantum *meruit* payment for their services to obtain planning permission.”
2. “Courts have taken a restrictive approach to the interpretation of such clauses to avoid unfair outcomes, analogous to their contra *proferentem* interpretation of wide clauses, excluding or limiting the liability for breach.”
3. “The plaintiffs were never in *delicto* [implicated in conscious wrongdoing] because they did not know the vital facts that would make the performance of the contract illegal.”

**Fig 3:**
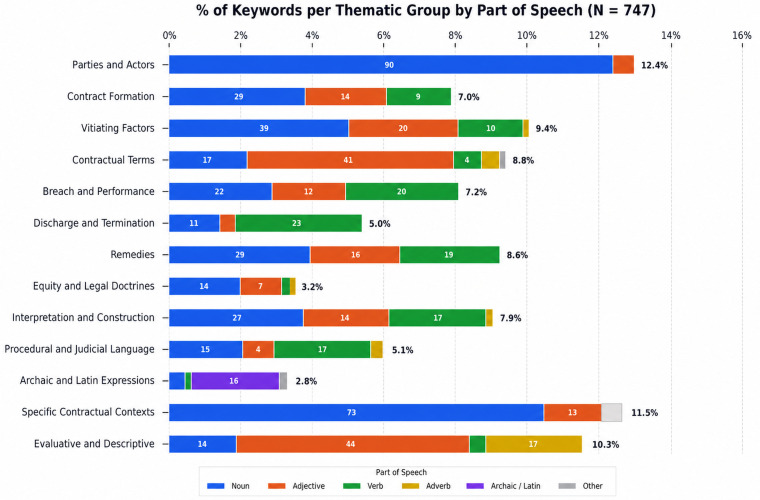
Distribution of grammatical forms across thematic groups.

The final step of the analysis involves the distribution of the grammatical forms pertaining to each thematic group. Fig 3 shows distinct variation in the use of these forms within thematic groups. Overall, categories on *parties and actors* and *contractual contexts* are characterized by the extensive presence of nominal structures which are needed in naming entities, describing concepts, and outlining contractual principles. Unsurprisingly, *evaluative and descriptive* group has the largest number of adjectival and adverbial forms. The co-occurrence of the two-word classes appear to mirror their role in specifying conditions, qualities, and legal states.

### Possible classroom intervention using the CLKL

The literature offers various techniques to help students expand their knowledge of domain-specific vocabulary. Items on the CLKL are presented as a keyness-informed, theme-based list. One particularly effective method is to present lexical items within a meaningful context. English for Legal Purposes educators may draw on the list for target keywords, creating multiple in-class activities as well as long-term projects. As an example, a lesson may begin with the instructor explaining a group of keywords on core contractual parties (e.g., acceptor, offeror, promisee). The discussion proceeds with the whole class giving illustrative statements exemplifying the role underlying each side in the contractual relationship. To enhance students’ ability to notice these vocabulary items, educators can employ several approaches, with the most common being textual enhancement [46,47]. This approach can be implemented by highlighting, underlining, or italicizing target vocabulary. It is argued [48] that unknown words are more easily learned if they are embedded within a familiar context. Contextualizing target words is particularly useful for learning legal concepts. Bhatia [49,50] argues that adequately interpreting several legal terms depends largely on the context in which they occur. Drawing on the CLKL, English for Legal Purposes educators may select vocabulary items and incorporate them within meaningful contexts to demonstrate their use in creating and interpreting legally binding contracts. Such an intervention is crucial for raising awareness among non-native speakers whose understanding of these key items may not yet approached a native-like level. To further facilitate use among non-native speakers, the keywords have been assigned the appropriate parts of speech (POS). POS-tagged items foster learning and instructional intervention by helping educators and materials designers create more targeted lessons and activities that focus on both the grammatical structure and the contextual usage, ultimately enabling students to better understand and apply knowledge of these terms within various legal discourse settings.

**Fig 4:**
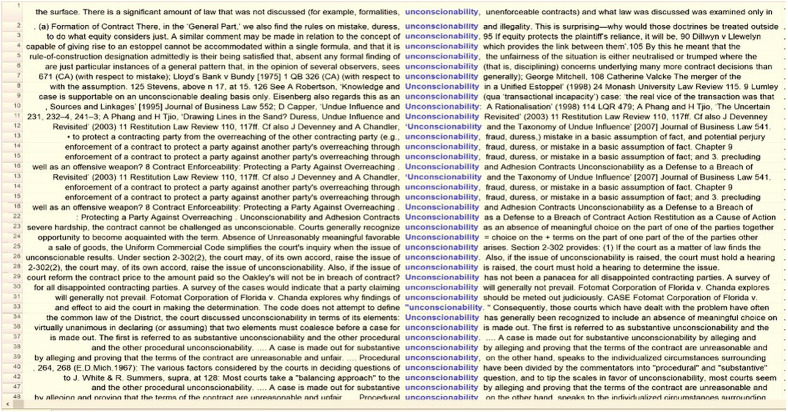
Concordance lines showing multiple occurrences of the keyword unconscionability.

Another instructional intervention involves careful note-taking. Jin and Webb [51] found that learners who write unfamiliar words in notes are more likely to learn those words than those who do not. Students may find several note-taking strategies useful, facilitating later revision. Hyland and Tse [52] argued that exposure to corpus-informed lists is useful for English for Academic Purposes (EAP) learners, provided that such lists were derived from the genres students will need to write and the texts they will need to read (p. 251). Once learners are presented with these lists, they can be guided to “notice” key vocabulary through repeated exposure and through temporary de-contextualization activities. The concordance lines generated by corpus tools provide a framework for the immediate environment of the target words, thus helping instructors approach the teaching of legal terms more effectively. By drawing on the study data, the word unconscionability occurs in different contexts and exhibits a linguistic association with specific words, such as fraud, as illustrated in Fig 4.

**Fig 5:**
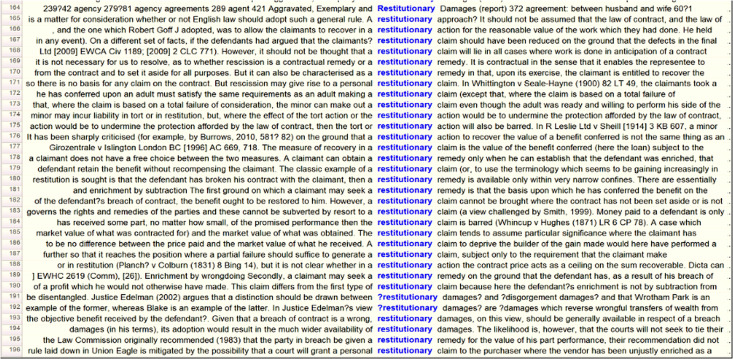
Concordance lines displaying the co-text features of the keyword restitutionary.

Fig 5 shows the concordance lines of the adjective restitutionary, which seems to modify a range of legal terms, such as damages, approaches, claims, and remedies. Instructors should be aware that these lines are just a selected group of sentences and that the corpus incorporates several example sentences in which the target word occurs.

To fully elucidate the nuanced meanings of the items on the list, instructors are advised to consult specialized dictionaries and subject-specific glossaries. For example, the word privity is given an elaborate treatment in Black’s Law Dictionary [53], defining the term and giving a detailed description of its sub-entries such as privity of contract. It is important to remember that not all keywords in the CLKL are given entries in standard dictionaries. Some keywords identified in the list are highly specialized legal terms specific to the law of contracts. Some others are recently coined, remaining uncodified lexicographically.

A final possible way to teach the items on the list is to apply Coxhead and Byrd’s [54] six-point procedure. First, students are encouraged to use their receptive vocabulary in productive tasks. An example of this point is to have them summarize the content of an imaginary contract and reproduce it in writing or as an oral presentation. Second, students are alerted to the differences between L1 and L2 writing systems. This step aims to raise awareness of the written form of the words. Third, technical vocabulary is rarely encountered in daily communication, implying that the students should make a deliberate effort to maximize learning by reading specialist terminology. Fourth, students need to expand their receptive and productive knowledge of specialist vocabulary that characterizes specific academic disciplines. Receptive knowledge facilitates comprehension of written and spoken materials, while productive knowledge enables students to use law-specific terms in both speech and writing.

Demonstrating thorough knowledge of and familiarity with the lexical fabric of a particular academic domain is key to better understanding and effective communication. Fifth, students must remember that learning the vocabulary of a specific discipline requires knowledge of a complex web of collocations, lexical bundles, phrases, and abbreviations. Finally, the real challenge for most students is the different methods by which a single word can be used. Developing awareness of and sensitivity to the various ways a word can be used is an objective underlying vocabulary-focused instruction.

## Conclusions

In response to the lexical demands of an increasing number of international students and foreign-trained law professionals, this study presents a 747-word list that aims to strengthen students’ knowledge of specialized lexis, deepen their understanding of expressions relevant to the law of contracts, and help them navigate texts of varying legal complexity. Through a combination of corpus treatment and keyness analysis, this study presents this list as a pedagogically useful resource for legal vocabulary. This study demonstrates how corpus tools and keyness analysis can be combined to produce targeted academic vocabulary suitable for classroom teaching. The findings may address some shortcomings in current legal education practices, such as ignoring discourse-relevant concepts [16], drawing on materials unsuitable for non-English-speaking audiences [3], and relying on extensive inauthentic content for promoting legal writing proficiency [31].

In conclusion, two important limitations are highlighted, and some directions for future research are offered. An important limitation lies in the discipline-specific nature of these keywords, restricting their potential use to the law of contracts. Modern English for Professional Purposes instruction, however, is expected to address a wide range of topics and professional domains. Another limitation is that the study draws on written textbooks, excluding other important genres such as memos, actual contracts, and court rulings. There are also some important pathways for future research. First, the results obtained from the corpus treatment and keyness analysis can be buttressed by opinions gleaned from disciplinary experts on matters related to the pedagogical value of corpus-derived patterns. Human intervention can help reduce the number of keywords, resulting in a more manageable and concise vocabulary list. Second, future researchers may also consider exploring the extent to which the items listed here can be found in specialized dictionaries and glossaries. Taken together, these two procedures can improve current research practices and help produce data-driven materials conducive to better learning.

## Supporting information

S1 Appendix List of textbooks used to build the study corpus. (DOCX)

S2 Appendix Contracts Law Keyword List (CLKL). (DOCX)
