# Supplementary material for: Developing a contracts law keyword list (CLKL) for academic legal education: A corpus-based, keyness-informed study
Source: PLoS One. 2026 Jul 6;21(7):e0352766. doi: 10.1371/journal.pone.0352766 (PMC13336195; doi:10.1371/journal.pone.0352766)
Supplement: S1 Appendix — (DOCX) [file pone.0352766.s001.docx]

# S1 Appendix

List of textbooks used to build the study corpus.

1. Andrews, N. (2011). Contract Law. Cambridge: Cambridge University Press.

2. Benson, P. (Ed.). (2001). The Theory of Contract Law: New Essays. Cambridge: Cambridge University Press.

3. Bix, B. (2013). Contract Law: Rules, Theory, and Context. Cambridge: Cambridge University Press.

4. Charman, M. (2007). Contract Law. Oregon: Willan Publishing.

5. Chen-Wishart, M. (2012). Contract Law (4th ed.). Oxford: Oxford University Press.

6. Eisenberg, M. & Gergen, M. (2018). Foundational Principles of Contract Law. Oxford: Oxford University Press.

7. Elliott, C. & Quinn, F. (2009). Contract Law (7th ed.). Harlow: Pearson Longman.

8. Furmston, M. P. (Ed.). (2020). The Future of the Law of Contract. London: Routledge.

9. Hevia, M. (2013). Reasonableness and Responsibility: A Theory of Contract Law. New York: Springer.

10. Hogg, M. (2011). Promises and Contract Law. Cambridge: Cambridge University Press.

11. Hook, M. (2016). The Choice of Law Contract. Oxford: Hart Publishing.

12. MacMillan, C. (2010). Mistakes in Contract Law. Oxford: Hart Publishing.

13. McKendrick, E. (2012). Contract Law: Text, Cases, and Materials (5th ed.). Oxford: Oxford University Press.

14. Monaghan, N. & Monaghan, C. (2013). Beginning Contract Law. London: Routledge.

15. Neyers, J., Bronaugh, R. & Pitel, S. (Eds.). (2009). Exploring Contract Law. Oxford: Hart Publishing.

16. O'Sullivan, J. & Hilliard, J. (2016). The Law of Contract. Oxford: Oxford University Press.

17. Paterson, J. & Robertson, A. (2012). Principles of Contract Law (4th ed.). Sydney: Thomson Reuters.

18. Stone, R. (2009). The Modern Law of Contract (8th ed.). London: Routledge-Cavendish.

19. Thampapillai, D., Tan, V. & Bozzi, C. (2012). Contract Law: Text and Cases. Oxford: Oxford University Press.

20. Young, M. (2009). Understanding Contract Law. London: Routledge.
