# Supplementary material for: Developing a contracts law keyword list (CLKL) for academic legal education: A corpus-based, keyness-informed study
Source: PLoS One. 2026 Jul 6;21(7):e0352766. doi: 10.1371/journal.pone.0352766 (PMC13336195; doi:10.1371/journal.pone.0352766)
Supplement: S2 Appendix — (DOCX) [file pone.0352766.s002.docx]

# A2 Appendix

**Contracts Law Keyword List (CLKL): 747 keywords arranged by keyness score**

| Rank | Keyword | POS | Frequency |
| --- | --- | --- | --- |
| 1 | privity | N | 711 |
| 2 | repudiation | N | 612 |
| 3 | unconscionability | N | 475 |
| 4 | restitutionary | Adj. | 445 |
| 5 | parol | Adj. | 374 |
| 6 | representee | N | 337 |
| 7 | oats | N | 298 |
| 8 | representor | N | 290 |
| 9 | rogue | N | 290 |
| 10 | disgorgement | N | 243 |
| 11 | repudiatory | Adj. | 209 |
| 12 | charterers | N. | 206 |
| 13 | third-party | Adj. | 203 |
| 14 | auctioneer | N | 199 |
| 15 | non-performance | N | 199 |
| 16 | donative | Adj. | 189 |
| 17 | vitiating | Adj. | 169 |
| 18 | innominate | Adj. | 167 |
| 19 | misapprehension | N | 163 |
| 20 | pre-existing | Adj. | 144 |
| 21 | hire-purchase | Adj. | 143 |
| 22 | charterparty | N | 138 |
| 23 | counter-offer | N | 137 |
| 24 | charterer | N | 136 |
| 25 | coronation | N | 134 |
| 26 | estopped | V | 133 |
| 27 | lading | N | 130 |
| 28 | obligor | N | 129 |
| 29 | executory | Adj. | 120 |
| 30 | (quantum) meruit | Archaic | 120 |
| 31 | non-disclosure | N | 118 |
| 32 | hirer | N | 116 |
| 33 | instalment | N | 112 |
| 34 | non-pecuniary | Adj. | 110 |
| 35 | necessaries | N | 106 |
| 36 | face-to-face | Adj. | 104 |
| 37 | unconscientious | Adj. | 104 |
| 38 | misstatement | N | 103 |
| 39 | boilerplate | N. & Adj. | 98 |
| 40 | non-contractual | Adj. | 98 |
| 41 | tanker | N | 97 |
| 42 | long-term | Adj. | 96 |
| 43 | payee | N. | 96 |
| 44 | promisees | N. | 95 |
| 45 | promisors | N. | 91 |
| 46 | surveyor | N. | 88 |
| 47 | well-known | Adj. | 88 |
| 48 | contract-breaker | N. | 86 |
| 49 | one-sided | Adj. | 86 |
| 50 | part-payment | Adj. | 85 |
| 51 | fraudster | N. | 84 |
| 52 | assents | N & V | 79 |
| 53 | shipowners | N. | 79 |
| 54 | assumpsit | V. | 77 |
| 55 | officious | Adj. | 76 |
| 56 | stevedores | N. | 75 |
| 57 | windfall | N. | 74 |
| 58 | vitiated | V. | 73 |
| 59 | peerless | Adj. | 72 |
| 60 | self-induced | N. | 72 |
| 61 | set-off | N. & V. | 72 |
| 62 | amenity | N. | 71 |
| 63 | pretences | N. | 68 |
| 64 | impropriety | N. | 66 |
| 65 | tow | N. & V. | 66 |
| 66 | attorney-general | N. | 65 |
| 67 | pre-estimate | N. & V. | 65 |
| 68 | disposals | N. | 64 |
| 69 | inconsistently | ADV. | 64 |
| 70 | non-compliance | N. | 64 |
| 71 | non-commercial | Adj. | 63 |
| 72 | repudiating | V., Adj. & N | 63 |
| 73 | decision-making | N. & Adj. | 58 |
| 74 | auctions | N. | 57 |
| 75 | defaulted | V. | 57 |
| 76 | well-being | N. | 57 |
| 77 | (contra) preferentem | Archaic | 56 |
| 78 | shaft | N. | 56 |
| 79 | acceptances | N. | 55 |
| 80 | reliance-based | Adj. | 54 |
| 81 | transparencies | N. | 54 |
| 82 | extravagant | Adj. | 53 |
| 83 | hemp | N. | 53 |
| 84 | novation | N. | 53 |
| 85 | vitiate | V. | 53 |
| 86 | shipowner | N. | 51 |
| 87 | sureties | N. | 51 |
| 88 | bargained-for | Adj. | 50 |
| 89 | well-established | Adj. | 50 |
| 90 | acceptor | N. | 49 |
| 91 | bailment | N. | 49 |
| 92 | self-interest | N. | 49 |
| 93 | severable | Adj. | 49 |
| 94 | transfield | N. | 49 |
| 95 | explicable | Adj. | 49 |
| 96 | non-fraudulent | Adj. | 49 |
| 97 | obligee | N. | 48 |
| 98 | refinery | N. | 48 |
| 99 | non-existence | N. | 46 |
| 100 | payor | N | 46 |
| 101 | estoppels | N. | 45 |
| 102 | shopkeeper | N. | 45 |
| 103 | improvident | Adj. | 44 |
| 104 | invoice | N. | 44 |
| 105 | merchantable | Adj. | 44 |
| 106 | non-mistaken | Adj. | 44 |
| 107 | subject-matter | N. | 44 |
| 108 | vis-a-vis | Prep. | 43 |
| 109 | non-existent | Adj. | 41 |
| 110 | amalgamated | Adj. | 40 |
| 111 | cross-purposes | N. | 40 |
| 112 | emptor | N. | 40 |
| 113 | impracticability | N. | 40 |
| 114 | booked | V. | 39 |
| 115 | breaker | N. | 39 |
| 116 | innocently | Adv. | 39 |
| 117 | overdraft | N. | 39 |
| 118 | referential | Adj. | 39 |
| 119 | sub-contractors | N. | 39 |
| 120 | vows | N. | 39 |
| 121 | advert | N. | 38 |
| 122 | lessees | N. | 38 |
| 123 | repudiates | V. | 38 |
| 124 | utterance | N. | 38 |
| 125 | equivocal | Adj. | 37 |
| 126 | forgo | V. | 37 |
| 127 | non-performing | Adj. | 37 |
| 128 | self-help | N. | 37 |
| 129 | subsisting | Adj. & V | 37 |
| 130 | unconscionably | Adv. | 37 |
| 131 | victimisation | N | 37 |
| 132 | would-be | Adj. | 37 |
| 133 | delicto (in pari delicto) | Archaic | 36 |
| 134 | falsity | N. | 36 |
| 135 | harshness | N. | 36 |
| 136 | howsoever | Pronoun | 36 |
| 137 | silicones | N. | 36 |
| 138 | cab | N. | 35 |
| 139 | misrepresentor | N. | 35 |
| 140 | overborne | V. | 35 |
| 141 | unenforceability | N | 35 |
| 142 | absurdity | N. | 34 |
| 143 | Culpa in contrahendo | Archaic | 34 |
| 144 | non-payment | N. | 34 |
| 145 | opportunism | N. | 34 |
| 146 | assented | V. | 33 |
| 147 | commonsense | N & Adj. | 33 |
| 148 | constable | N. | 33 |
| 149 | disgorge | V. | 33 |
| 150 | non-reliance | N. | 33 |
| 151 | puff | N. | 33 |
| 152 | resile | V. | 33 |
| 153 | self-service | Adj. | 33 |
| 154 | architect | N. | 32 |
| 155 | feveroles | N. | 32 |
| 156 | non-delivery | N. | 32 |
| 157 | offerees | N. | 32 |
| 158 | peppercorn | N. | 32 |
| 159 | shipper | N. | 32 |
| 160 | sub-contractor | N. | 32 |
| 161 | undervalue | N. | 32 |
| 162 | unreasonableness | N. | 32 |
| 163 | unsupported | V & Adj. | 32 |
| 164 | assenting | V. | 32 |
| 165 | illegitimacy | N. | 32 |
| 166 | long-standing | Adj. | 32 |
| 167 | pre-contract | Adj. | 32 |
| 168 | providence | N. | 32 |
| 169 | referable | Adj. | 31 |
| 170 | second-hand | Adj. | 31 |
| 171 | three-party | Adj. | 31 |
| 172 | unexpressed | Adj. | 31 |
| 173 | acquiesced | V. | 31 |
| 174 | advantage-taking | Adj. | 31 |
| 175 | fiduciaries | N. | 30 |
| 176 | horsebeans | N. | 30 |
| 177 | non-binding | Adj. | 30 |
| 178 | reposed | V. | 30 |
| 179 | vow | N. | 30 |
| 180 | franchisee | N. | 30 |
| 181 | irrecoverable | Adj. | 29 |
| 182 | mischief | N. | 29 |
| 183 | oust | V. | 29 |
| 184 | self-evident | Adj. | 29 |
| 185 | trawler | N. | 29 |
| 186 | trawlers | N. | 29 |
| 187 | unperformed | Adj. | 29 |
| 188 | carpentry | N. | 29 |
| 189 | conveyancing | Adj. | 28 |
| 190 | open-ended | Adj. | 28 |
| 191 | renege | V. | 28 |
| 192 | rewriting | N. | 28 |
| 193 | vitiation | N | 28 |
| 194 | demolition | N. | 28 |
| 195 | equities | N. | 28 |
| 196 | extortionate | Adj. | 28 |
| 197 | non-monetary | Adj. | 28 |
| 198 | one-off | Adj. | 27 |
| 199 | unsigned | Adj. | 27 |
| 200 | administratrix | N. | 27 |
| 201 | barges | N. | 26 |
| 202 | desertion | N. | 26 |
| 203 | guineas | N. | 26 |
| 204 | prepayment | N. | 26 |
| 205 | prohibitory | Adj. | 26 |
| 206 | rebutting | N. | 26 |
| 207 | retraction | N. | 26 |
| 208 | signer | N. | 26 |
| 209 | supposition | N. | 26 |
| 210 | tankships | N. | 26 |
| 211 | valuers | N. | 26 |
| 212 | ascendancy | N. | 26 |
| 213 | defraud | N. | 25 |
| 214 | dishonestly | Adv. | 25 |
| 215 | far-reaching | Adj. | 25 |
| 216 | hedges | N. | 25 |
| 217 | liquidators | N. | 25 |
| 218 | out-of-pocket | Adj. | 25 |
| 219 | pre-contractual | Adj. | 25 |
| 220 | rectifying | N. | 25 |
| 221 | undo | V. | 25 |
| 222 | unsolicited | Adj. | 25 |
| 223 | coach | N. | 24 |
| 224 | counter-performance | N. | 24 |
| 225 | covenanted | V. | 24 |
| 226 | ecclesiastical | Adj. | 24 |
| 227 | helpfully | Adv. | 24 |
| 228 | Hypothesi | N. | 24 |
| 229 | judge-made | Adj. | 24 |
| 230 | meritorious | Adj. | 24 |
| 231 | receivers | N. | 24 |
| 232 | recompense | V. | 24 |
| 233 | rewrite | V. | 24 |
| 234 | sulphur | N. | 24 |
| 235 | adhesion | N. | 24 |
| 236 | demolish | V. | 24 |
| 237 | (res) extincta | N. | 24 |
| 238 | inessential | Adj. | 24 |
| 239 | intimation | N. | 24 |
| 240 | literalism | N. | 24 |
| 241 | opportunistically | Adv. | 23 |
| 242 | (inter) praesentes | Archaic | 23 |
| 243 | redelivery | N. | 23 |
| 244 | renegotiate | V. | 23 |
| 245 | seaworthiness | N. | 23 |
| 246 | self-imposed | Adj. | 23 |
| 247 | assignable | Adj. | 23 |
| 248 | bailee | N. | 23 |
| 249 | cautionary | Adj. | 23 |
| 250 | counterclaimed | V. | 23 |
| 251 | galleries | N. | 23 |
| 252 | impute | V. | 22 |
| 253 | intoxication | N. | 22 |
| 254 | non-conforming | Adj. | 22 |
| 255 | non-essential | Adj. | 22 |
| 256 | promise-based | Adj. | 22 |
| 257 | (jus) quaesitum (tertio) | Archaic | 22 |
| 258 | sentimental | Adj. | 22 |
| 259 | subtracting | V. | 22 |
| 260 | widgets | N. | 22 |
| 261 | cross-border | V. & Adj. | 22 |
| 262 | dishonoured | V. | 22 |
| 263 | festival | N. | 22 |
| 264 | foundries | N. | 22 |
| 265 | free-standing | Adj. | 22 |
| 266 | honoured | V. | 22 |
| 267 | notional | Adj. | 21 |
| 268 | part-performance | N. | 21 |
| 269 | puffs | N. | 21 |
| 270 | two-party | Adj. | 21 |
| 271 | adduce | V. | 21 |
| 272 | blackmail | N. | 21 |
| 273 | cabbage | N. | 21 |
| 274 | cancelling | V. | 21 |
| 275 | case-by-case | N. | 21 |
| 276 | clear-cut | Adj. | 21 |
| 277 | counter-claim | N. | 21 |
| 278 | dealership | N. | 21 |
| 279 | detrimentally | Adv. | 21 |
| 280 | non-promissory | N. | 21 |
| 281 | pre-nuptial | Adj. | 21 |
| 282 | proffered | V. | 21 |
| 283 | two-sided | Adj. | 20 |
| 284 | unavailability | N. | 20 |
| 285 | unliquidated | Adj. | 20 |
| 286 | unload | V. | 20 |
| 287 | vending | N. | 20 |
| 288 | winnings | N. | 20 |
| 289 | apprenticeship | N. | 20 |
| 290 | carpet | N. | 20 |
| 291 | cashier | N. | 20 |
| 292 | disclaim | V. | 20 |
| 293 | draftsman | N. | 20 |
| 294 | extra-judicially | Adv. | 20 |
| 295 | indistinguishable | Adj. | 20 |
| 296 | invalidates | V. | 20 |
| 297 | offerors | N. | 20 |
| 298 | one-third | N. | 20 |
| 299 | paragon | N. | 19 |
| 300 | salesman | N. | 19 |
| 301 | subcontract | N. | 19 |
| 302 | suspensory | Adj. | 19 |
| 303 | tear-off | Adj. | 19 |
| 304 | tenderer | N. | 19 |
| 305 | unbeknown | Adj. | 19 |
| 306 | adventitious | Adj. | 18 |
| 307 | axioms | N. | 18 |
| 308 | beware | V. | 18 |
| 309 | bother | N. | 18 |
| 310 | carpenters | N. | 18 |
| 311 | coupon | N. | 18 |
| 312 | defrauded | Adj. & V. | 18 |
| 313 | demised | Adj. | 18 |
| 314 | eventuate | V. | 18 |
| 315 | evinced | V. | 18 |
| 316 | formalist | Adj. | 18 |
| 317 | halfway | Adv. | 18 |
| 318 | obscurity | N. | 18 |
| 319 | pactum | Archaic | 18 |
| 320 | proferens | Archaic | 18 |
| 321 | remediable | Adj. | 18 |
| 322 | renegotiated | V. & Adj. | 18 |
| 323 | repayments | N. | 18 |
| 324 | subvert | V. | 18 |
| 325 | terminable | Adj. | 18 |
| 326 | terrorem | Archaic | 18 |
| 327 | vitiates | V. | 18 |
| 328 | voidness | N. | 18 |
| 329 | alienable | Adj. | 17 |
| 330 | auctioneers | N. | 17 |
| 331 | bales | N. | 17 |
| 332 | charterparties | N. | 17 |
| 333 | consignor | N. | 17 |
| 334 | courier | N. | 17 |
| 335 | cut-off | Adj. | 17 |
| 336 | drunkenness | N. | 17 |
| 337 | gloss | N. | 17 |
| 338 | illiteracy | N. | 17 |
| 339 | improvidence | N. | 17 |
| 340 | legatees | N. | 17 |
| 341 | mortgagee | N. | 17 |
| 342 | nod | N. | 17 |
| 343 | non-legal | Adj. | 17 |
| 344 | non-occurrence | N. | 17 |
| 345 | obviates | V. | 17 |
| 346 | remitted | V. | 17 |
| 347 | requisitioned | V. | 17 |
| 348 | rigour | N. | 17 |
| 349 | self-interested | Adj. | 17 |
| 350 | sewer | N. | 17 |
| 351 | unacceptably | Adv. | 17 |
| 352 | unaccepted | Adj. | 17 |
| 353 | uneasily | Adv. | 17 |
| 354 | utterances | N. | 17 |
| 355 | wary | Adj. | 17 |
| 356 | wide-ranging | Adj. | 17 |
| 357 | workmanship | N. | 17 |
| 358 | consignee | N. | 16 |
| 359 | countenance | N. & V. | 16 |
| 360 | counter-promise | N. | 16 |
| 361 | defectively | Adv. | 16 |
| 362 | demolishing | V. | 16 |
| 363 | demurrer | N | 16 |
| 364 | deserters | N. | 16 |
| 365 | disincentives | N. | 16 |
| 366 | dominions | N. | 16 |
| 367 | drains | N. | 16 |
| 368 | exacting | Adj. | 16 |
| 369 | foregone | V. | 16 |
| 370 | gain-based | Adj. | 16 |
| 371 | lawn | N. | 16 |
| 372 | non-consumer | N. | 16 |
| 373 | promise-keeping | N. | 16 |
| 374 | simultaneity | N. | 16 |
| 375 | strand | N. | 16 |
| 376 | trainer | N. | 16 |
| 377 | unmeritorious | Adj. | 16 |
| 378 | vexation | N. | 16 |
| 379 | weighty | Adj. | 16 |
| 380 | well-informed | Adj. | 16 |
| 381 | all-or-nothing | N. | 15 |
| 382 | amity | N. | 15 |
| 383 | beads | N. | 15 |
| 384 | bets | N. | 15 |
| 385 | business-to-business | N. | 15 |
| 386 | cloakroom | N. | 15 |
| 387 | collieries | N. | 15 |
| 388 | committal | N. | 15 |
| 389 | conferment | N. | 15 |
| 390 | disprove | V. | 15 |
| 391 | (ex) gratia | Archaic | 15 |
| 392 | guarantors | N. | 15 |
| 393 | inequity | N. | 15 |
| 394 | irrebuttable | Adj. | 15 |
| 395 | joinder | N. | 15 |
| 396 | nodes | N. | 15 |
| 397 | non-breaching | Adj. | 15 |
| 398 | optimism | N. | 15 |
| 399 | overreaching | Adj. | 15 |
| 400 | penalise | V. | 15 |
| 401 | post-nuptial | Adj. | 15 |
| 402 | pre-emptive | Adj. | 15 |
| 403 | racehorse | N. | 15 |
| 404 | rescinds | V. | 15 |
| 405 | retracted | V. | 15 |
| 406 | surveyors | N. | 15 |
| 407 | tempered | V. | 15 |
| 408 | tenderers | N. | 15 |
| 409 | trifling | Adj. | 15 |
| 410 | uttered | V. | 15 |
| 411 | promisee | N. | 2,192 |
| 412 | estoppel | N. | 2,280 |
| 413 | promissory | Adj. | 1,286 |
| 414 | promisor | N. | 2,000 |
| 415 | unconscionable | Adj. | 797 |
| 416 | frustration | N. | 1,156 |
| 417 | misrepresentation | N. | 5,063 |
| 418 | duress | N. | 2,653 |
| 419 | offeror | N. | 1,857 |
| 420 | rescission | N. | 1,004 |
| 421 | frustrated | V. & Adj. | 637 |
| 422 | offeree | N. | 910 |
| 423 | claimant | N. | 3,274 |
| 424 | vendor | N. | 583 |
| 425 | unfairness | N. | 386 |
| 426 | misrepresentations | N. | 3,274 |
| 427 | repudiated | V. | 583 |
| 428 | remoteness | N. | 304 |
| 429 | extrinsic | Adj. | 181 |
| 430 | rescind | V. | 384 |
| 431 | anticipatory | Adj. | 195 |
| 432 | landlord | N. | 362 |
| 433 | frauds | N. | 236 |
| 434 | contemplation | N. | 251 |
| 435 | unenforceable | Adj. | 591 |
| 436 | deceit | N. | 230 |
| 437 | instalments | N. | 211 |
| 438 | builder | N. | 248 |
| 439 | supervening | Adj. | 132 |
| 440 | bystander | N. | 117 |
| 441 | tenders | N. | 143 |
| 442 | frustrating | Adj. | 214 |
| 443 | rescinded | V. | 184 |
| 444 | warranties | N. | 192 |
| 445 | solicitor | N. | 432 |
| 446 | restatement | N. | 460 |
| 447 | tenant | N. | 402 |
| 448 | undue | Adj. | 1,684 |
| 449 | voidable | Adj. | 485 |
| 450 | impliedly | Adv. | 207 |
| 451 | rectification | N. | 429 |
| 452 | untrue | Adj. | 118 |
| 453 | nominees | N. | 87 |
| 454 | appellants | N. | 257 |
| 455 | frustrate | V. | 145 |
| 456 | construing | V. | 117 |
| 457 | inducement | N. | 199 |
| 458 | warranty | N. | 683 |
| 459 | quantum | N. | 168 |
| 460 | gratuitous | Adj. | 344 |
| 461 | irrevocable | Adj. | 112 |
| 462 | rectified | V. | 78 |
| 463 | privy | Adj. | 361 |
| 464 | relational | Adj. | 270 |
| 465 | instantaneous | Adj. | 99 |
| 466 | forfeiture | N. | 185 |
| 467 | breach | N. | 7,630 |
| 468 | putative | Adj. | 183 |
| 469 | auction | N. | 274 |
| 470 | tenancy | N. | 66 |
| 471 | innocent | Adj. | 1,256 |
| 472 | contractual | Adj. | 4,473 |
| 473 | timber | N. | 368 |
| 474 | solicitors | N. | 106 |
| 475 | matrimonial | Adj. | 194 |
| 476 | onerous | Adj. | 176 |
| 477 | illusory | Adj. | 252 |
| 478 | purporting | V. | 111 |
| 479 | purported | Adj. | 87 |
| 480 | vendors | N. | 265 |
| 481 | foreseeability | N. | 132 |
| 482 | repudiate | V. | 84 |
| 483 | fraudulently | Adv. | 122 |
| 484 | forbearance | N. | 142 |
| 485 | breaching | N. & Adj. | 195 |
| 486 | tenants | N. | 221 |
| 487 | inequitable | Adj. | 130 |
| 488 | treatises | N. | 83 |
| 489 | contractually | Adv. | 145 |
| 490 | wedding | N. | 51 |
| 491 | actionable | Adj. | 227 |
| 492 | restitution | N. | 793 |
| 493 | unilateral | Adj. | 1,064 |
| 494 | misrepresented | V. | 47 |
| 495 | enforceability | N. | 336 |
| 496 | consequential | Adj. | 227 |
| 497 | aggrieved | Adj. | 424 |
| 498 | enforceable | Adj. | 1,312 |
| 499 | procured | V. | 126 |
| 500 | chartered | V. & Adj. | 90 |
| 501 | rebutted | V. | 152 |
| 502 | dishonesty | N. | 49 |
| 503 | axiomatic | Adj. | 44 |
| 504 | causation | N. | 261 |
| 505 | illegitimate | Adj. | 381 |
| 506 | reasonableness | N. | 598 |
| 507 | salvage | V. & Adj. | 106 |
| 508 | carelessness | N. | 61 |
| 509 | appellant | N. | 324 |
| 510 | cruise | N. | 56 |
| 511 | unhelpful | Adj. | 40 |
| 512 | void | Adj. | 1,235 |
| 513 | fraudulent | Adj. | 413 |
| 514 | indemnity | N. | 171 |
| 515 | (ab) initio | Archaic | 89 |
| 516 | equitable | Adj. | 1,575 |
| 517 | booking | N. | 37 |
| 518 | construe | V. | 66 |
| 519 | dissented | V. | 880 |
| 520 | lease | N. | 91 |
| 521 | orthodox | Adj. | 708 |
| 522 | detriment | N. | 708 |
| 523 | pleaded | V. | 116 |
| 524 | presumptively | Adv. | 36 |
| 525 | impugned | V. | 68 |
| 526 | obiter | N. | 109 |
| 527 | fiduciary | Adj. | 372 |
| 528 | breached | V. | 546 |
| 529 | renunciation | N. | 100 |
| 530 | endeavours | N. | 92 |
| 531 | tendering | Adj. & N. | 44 |
| 532 | warehouse | N. | 54 |
| 533 | rescinding | N. | 37 |
| 534 | arrears | N. | 47 |
| 535 | defendants | N. | 47 |
| 536 | intelligible | Adj. | 1,613 |
| 537 | apportion | V. | 33 |
| 538 | unequivocal | Adj. | 83 |
| 539 | guarantor | N. | 89 |
| 540 | contemplated | V. | 304 |
| 541 | purports | V. | 152 |
| 542 | payable | Adj. | 437 |
| 543 | doctrines | N. | 558 |
| 544 | onus | N. | 155 |
| 545 | factum | N. | 122 |
| 546 | sue | V. | 900 |
| 547 | rectify | V | 87 |
| 548 | assignee | N. | 209 |
| 549 | claimants | N. | 283 |
| 550 | inconvenience | N. | 174 |
| 551 | tacit | Adj. | 135 |
| 552 | revocable | Adj. | 38 |
| 553 | bidders | N. | 35 |
| 554 | transaction | N. | 2,096 |
| 555 | liquidated | Adj. | 222 |
| 556 | tendered | V. | 66 |
| 557 | dicta | Archaic | 62 |
| 558 | bidder | N. | 161 |
| 559 | reprehensible | Adj. | 33 |
| 560 | assent | N. | 293 |
| 561 | plaintiffs | N. | 1,327 |
| 562 | appeal | N. | 3,075 |
| 563 | treatise | N. | 258 |
| 564 | subjectively | Adv. | 365 |
| 565 | rebut | V. | 80 |
| 566 | awarded | V. | 667 |
| 567 | leases | N. | 124 |
| 568 | entitle | V. | 158 |
| 569 | imbalance | N. | 170 |
| 570 | suing | N. | 115 |
| 571 | indemnify | V. | 73 |
| 572 | discharged | V. | 292 |
| 573 | proposition | N. | 523 |
| 574 | invalidating | Adj. & V. | 41 |
| 575 | collateral | N. | 370 |
| 576 | creditworthiness | N. | 38 |
| 577 | wrongdoing | N. | 134 |
| 578 | lender | N. | 227 |
| 579 | defendant | N. | 4,950 |
| 580 | disadvantage | N. | 335 |
| 581 | exempting | Adj. | 57 |
| 582 | unforeseeable | Adj. | 52 |
| 583 | stipulation | N. | 237 |
| 584 | award | N. | 961 |
| 585 | purport | V. | 129 |
| 586 | absurd | Adj. | 73 |
| 587 | affirmation | N. | 102 |
| 588 | coerced | V. | 80 |
| 589 | pleading | N. | 961 |
| 590 | affection | N. | 129 |
| 591 | discharge | V. | 490 |
| 592 | negotiate | V. | 480 |
| 593 | assignor | N. | 117 |
| 594 | occasioned | V. | 51 |
| 595 | exposition | N. | 67 |
| 596 | (per) annum | Archaic | 46 |
| 597 | normatively | Adv. | 55 |
| 598 | injustice | N. | 241 |
| 599 | taxonomy | N. | 50 |
| 600 | characterisation | N. | 68 |
| 601 | freight | N. | 43 |
| 602 | contingent | Adj. | 273 |
| 603 | propositions | N. | 248 |
| 604 | expenditure | N. | 320 |
| 605 | unreasonable | Adj. | 575 |
| 606 | sanctity | N. | 37 |
| 607 | suffices | V. | 60 |
| 608 | doctrine | N. | 3,492 |
| 609 | breaches | V. | 476 |
| 610 | bidding | N. | 55 |
| 611 | equity | N. | 1,676 |
| 612 | trustee | N. | 215 |
| 613 | exceptionally | Adv. | 114 |
| 614 | extinguish | V. | 45 |
| 615 | apportionment | N. | 51 |
| 616 | indifferent | Adj. | 53 |
| 617 | trivial | Adj. | 110 |
| 618 | plaintiff | N. | 3,942 |
| 619 | proprietary | Adj. | 334 |
| 620 | fulfil | V. | 88 |
| 621 | oppressive | Adj. | 64 |
| 622 | sensibly | Adv. | 46 |
| 623 | entitling | V. | 42 |
| 624 | incapacity | N. | 195 |
| 625 | rebuttable | Adj. | 55 |
| 626 | plead | V. | 53 |
| 627 | readiness | N. | 49 |
| 628 | nominal | Adj. | 226 |
| 629 | renegotiation | N. | 48 |
| 630 | contributory | Adj. | 165 |
| 631 | sues | V. | 44 |
| 632 | admissible | Adj. | 135 |
| 633 | materiality | N. | 51 |
| 634 | immaterial | Adj. | 77 |
| 635 | transactional | Adj. | 103 |
| 636 | affirm | V. | 75 |
| 637 | insisting | N. | 58 |
| 638 | ascertaining | V. | 86 |
| 639 | wrongfully | Adv. | 71 |
| 640 | harsh | Adj. | 125 |
| 641 | caveat | N. | 52 |
| 642 | unwillingness | N. | 61 |
| 643 | criticised | V. | 164 |
| 644 | enrichment | N. | 437 |
| 645 | defaulting | Adj. | 44 |
| 646 | incurring | V. | 62 |
| 647 | criticisms | N. | 105 |
| 648 | usages | N. | 47 |
| 649 | fraud | N. | 925 |
| 650 | minus | N. & Adj. | 47 |
| 651 | extortion | N. | 47 |
| 652 | utmost | Adj. | 117 |
| 653 | trespass | V. | 48 |
| 654 | voluntariness | N. | 53 |
| 655 | doctrinal | Adj. | 203 |
| 656 | mercantile | Adj. | 63 |
| 657 | revoke | V. | 136 |
| 658 | brokers | N. | 56 |
| 659 | surety | N. | 190 |
| 660 | insert | V. | 59 |
| 661 | bid | V. & N. | 350 |
| 662 | subjective | Adj. | 599 |
| 663 | cancelled | V. | 90 |
| 664 | suffice | V. | 146 |
| 665 | developer | N. | 66 |
| 666 | incur | V. | 128 |
| 667 | matrix | N. | 77 |
| 668 | discount | N. & V. | 62 |
| 669 | seal | N. | 109 |
| 670 | metropolitan | Adj. | 73 |
| 671 | sued | V. | 650 |
| 672 | negotiations | N. | 800 |
| 673 | manifested | V. | 127 |
| 674 | contemplate | V. | 127 |
| 675 | dictum | Archaic | 127 |
| 676 | mitigation | N. | 279 |
| 677 | unexpected | Adj. | 144 |
| 678 | insistence | N. | 56 |
| 679 | fusion | N. | 78 |
| 680 | cancel | V. | 113 |
| 681 | mirror | Adj. | 70 |
| 682 | conveyance | N. | 54 |
| 683 | remedial | Adj. | 235 |
| 684 | incurred | V. | 445 |
| 685 | lenders | N. | 60 |
| 686 | mutuality | N. | 110 |
| 687 | overruled | V. | 58 |
| 688 | conceded | V. | 56 |
| 689 | accrued | V. | 89 |
| 690 | unsatisfactory | Adj. | 95 |
| 691 | convey | V. | 104 |
| 692 | overt | Adj. | 55 |
| 693 | ascertained | V. | 108 |
| 694 | lapse | V. | 116 |
| 695 | oral | Adj. | 541 |
| 696 | goods | N. | 4,263 |
| 697 | acquiescence | N. | 59 |
| 698 | exerted | V. | 79 |
| 699 | covenants | N. | 90 |
| 700 | efficacy | N. | 126 |
| 701 | (prima) facie | Archaic | 220 |
| 702 | (in) lieu | Archaic | 110 |
| 703 | mortgage | N. | 214 |
| 704 | substantively | Adv. | 65 |
| 705 | plea | N. | 145 |
| 706 | formalities | N. | 165 |
| 707 | negligent | Adj. | 165 |
| 708 | manifestly | Adv. | 67 |
| 709 | deceptive | Adj. | 198 |
| 710 | usage | N. | 206 |
| 711 | awarding | V. | 90 |
| 712 | reconcile | V. | 88 |
| 713 | deliberately | Adv. | 174 |
| 714 | negotiation | N. | 265 |
| 715 | tortious | Adj. | 138 |
| 716 | subsection | N. | 267 |
| 717 | negotiated | V. | 248 |
| 718 | exemplified | V. | 64 |
| 719 | defective | Adj. | 405 |
| 720 | negotiating | V. | 204 |
| 721 | waived | V. | 102 |
| 722 | detrimental | Adj. | 191 |
| 723 | weaker | Adj. | 138 |
| 724 | lessor | N. | 170 |
| 725 | entitled | V. | 1,946 |
| 726 | executed | V. | 303 |
| 727 | compel | V. | 171 |
| 728 | maxim | N. | 84 |
| 729 | negligence | N. | 996 |
| 730 | insurer | N. | 192 |
| 731 | entitlement | N. | 274 |
| 732 | render | V. | 455 |
| 733 | manifest | V. & Adj. | 140 |
| 734 | executor | N. | 70 |
| 735 | waive | V. | 78 |
| 736 | foreseeable | Adj. | 249 |
| 737 | tort | N. | 1,031 |
| 738 | disclaimer | N. | 80 |
| 739 | expressly | Adv. | 777 |
| 740 | cargo | N. | 209 |
| 741 | withdraw | V. | 232 |
| 742 | renders | V. | 161 |
| 743 | donee | N. | 130 |
| 744 | cancellation | N. | 88 |
| 745 | defects | N. | 296 |
| 746 | construed | V. | 270 |
| 747 | evidentiary | Adj. | 87 |

**Thematic Classification into groups and sub-groups (N = 747)**

| Group 1: Parties and Actors n = 93 · 12.4% of CLKL | | |  |
| --- | --- | --- | --- |
| Sub-category | n | Keywords (alphabetical) | |
| Commercial and Trade | 18 | auctioneer, auctioneers, bidder, bidders, brokers, builder, charterer, charterers, developer, franchisee, shipowner, shipowners, shipper, stevedores, sub-contractor, sub-contractors, tenderer, tenderers | |
| Other Professionals | 18 | administratrix, architect, attorney-general, cashier, consignee, consignor, draftsman, liquidators, receivers, salesman, shopkeeper, signer, surveyor, surveyors, three-party, trainer, two-party, valuers | |
| Core Contractual Parties | 16 | acceptor, contract-breaker, fraudster, obligee, obligor, offeree, offerees, offeror, offerors, payee, payor, promisee, promisees, promisor, promisors, rogue | |
| Property and Finance | 15 | bailee, hirer, insurer, landlord, legatees, lender, lenders, lessees, lessor, mortgagee, non-consumer, tenant, tenants, vendor, vendors | |
| Dispute Resolution | 14 | appellant, appellants, claimant, claimants, constable, defendant, defendants, misrepresentor, plaintiff, plaintiffs, representee, representor, solicitor, solicitors | |
| Third-Party and Fiduciary | 12 | assignee, assignor, donee, executor, fiduciaries, guarantor, guarantors, nominees, sureties, surety, third-party, trustee | |
| Group total | 93 | — | |

| Group 2: Specific Contractual Contexts n = 92 · 12.3% of CLKL |
| --- |

| Sub-category | n | Keywords (alphabetical) |
| --- | --- | --- |
| Maritime and Carriage | 17 | bales, barges, cargo, chartered, charterparties, charterparty, freight, lading, mercantile, salvage, shaft, tanker, tankships, tow, transfield, trawler, trawlers |
| Commerce and Finance | 17 | amalgamated, auction, auctions, bidding, booked, booking, cab, cloakroom, coach, cross-border, cruise, dealership, hire-purchase, invoice, overdraft, self-service, vending |
| Property and Conveyancing | 16 | convey, conveyance, conveyancing, creditworthiness, demised, dominions, drains, lawn, lease, leases, metropolitan, mortgage, sewer, strand, tenancy, warehouse |
| Goods and Commodities | 15 | beads, bets, cabbage, carpet, coupon, feveroles, goods, hedges, hemp, horsebeans, nodes, oats, racehorse, widgets, winnings |
| Events and Social Contexts | 14 | apprenticeship, coronation, courier, deserters, ecclesiastical, festival, galleries, guineas, matrimonial, post-nuptial, pre-nuptial, providence, requisitioned, wedding |
| Construction and Industry | 13 | carpenters, carpentry, collieries, demolish, demolishing, demolition, foundries, refinery, silicones, subcontract, sulphur, timber, workmanship |
| Group total | 92 | — |

| Group 3: Evaluative and Descriptive Language n = 77 · 10.3% of CLKL |
| --- |

| Sub-category | n | Keywords (alphabetical) |
| --- | --- | --- |
| Degree, Manner and Scope | 19 | defectively, deliberately, exceptionally, far-reaching, halfway, helpfully, immaterial, inconsistently, innocently, manifestly, normatively, sensibly, substantively, trifling, trivial, unacceptably, unexpected, utmost, wide-ranging |
| Legal Quality and Merit | 14 | clear-cut, explicable, indistinguishable, long-standing, meritorious, paragon, peerless, rigour, unmeritorious, unsupported, weighty, well-established, well-informed, well-known |
| Negative Moral Evaluation | 13 | dishonestly, extravagant, fraudulently, harsh, inequitable, negligent, officious, one-sided, opportunistically, oppressive, reprehensible, self-interested, wrongfully |
| Fairness and Reasonableness | 12 | absurd, absurdity, amity, cautionary, disadvantage, harshness, injustice, reasonableness, self-evident, unfairness, unreasonable, unreasonableness |
| Party Psychological State | 11 | aggrieved, equivocal, indifferent, innocent, optimism, sentimental, tempered, uneasily, unwillingness, wary, weaker |
| Situational and Policy Descriptors | 8 | adventitious, beware, bother, disincentives, exacting, unbeknown, unhelpful, unsatisfactory |
| Group total | 77 | — |

| Group 4: Vitiating Factors n = 70 · 9.4% of CLKL |
| --- |

| Sub-category | n | Keywords (alphabetical) |
| --- | --- | --- |
| Misrepresentation | 15 | actionable, carelessness, deceit, deceptive, falsity, inducement, misrepresentation, misrepresentations, misrepresented, misstatement, negligence, non-fraudulent, non-reliance, pretences, untrue |
| Undue Influence and Unconscionability | 15 | advantage-taking, ascendancy, extortionate, imbalance, improvidence, improvident, inequity, non-disclosure, reposed, unconscientious, unconscionability, unconscionable, unconscionably, undervalue, undue |
| Fraud and Dishonesty | 12 | defraud, defrauded, dishonesty, fraud, frauds, fraudulent, illegitimacy, illegitimate, impropriety, opportunism, procured, wrongdoing |
| Vitiation and Legal Consequences | 10 | invalidates, invalidating, vitiate, vitiated, vitiates, vitiating, vitiation, void, voidable, voidness |
| Duress | 9 | blackmail, coerced, duress, exerted, extortion, overborne, overreaching, self-interest, victimisation |
| Mistake and Incapacity | 9 | cross-purposes, drunkenness, illiteracy, incapacity, intoxication, misapprehension, non-existence, non-existent, non-mistaken |
| Group total | 70 | — |

| Group 5: Contractual Terms n = 66 · 8.8% of CLKL |
| --- |

| Sub-category | n | Keywords (alphabetical) |
| --- | --- | --- |
| Character and Attributes of Terms | 20 | endeavours, free-standing, illusory, inessential, long-term, merchantable, non-commercial, non-contractual, non-essential, non-legal, one-off, onerous, open-ended, pre-emptive, seaworthiness, second-hand, self-imposed, subject-matter, subsisting, would-be |
| Classification of Terms | 18 | collateral, contingent, covenanted, covenants, executory, expressly, impliedly, innominate, non-promissory, oral, parol, stipulation, tacit, unexpressed, usage, usages, warranties, warranty |
| Exclusion and Disclaimer | 10 | boilerplate, cut-off, disclaim, disclaimer, exempting, howsoever, insert, oust, prohibitory, tear-off |
| Validity and Enforceability | 10 | alienable, assignable, enforceability, enforceable, irrevocable, non-binding, severable, terminable, unenforceability, unenforceable |
| Contract Framework and Structure | 8 | adhesion, business-to-business, contractual, contractually, relational, transactional, two-sided, unilateral |
| Group total | 66 | — |

| Group 6: Remedies n = 64 · 8.6% of CLKL |
| --- |

| Sub-category | n | Keywords (alphabetical) |
| --- | --- | --- |
| Damages: Types and Quantification | 21 | apportion, apportionment, consequential, discount, expenditure, incur, incurred, incurring, liquidated, minus, nominal, non-pecuniary, one-third, out-of-pocket, penalise, pre-estimate, quantum, remitted, repayments, subtracting, unliquidated |
| Entitlement and Specific Remedies | 19 | award, awarded, awarding, compel, disposals, entitle, entitled, entitlement, entitling, indemnify, indemnity, irrecoverable, rectification, rectified, rectify, rectifying, remediable, remedial, self-help |
| Damages: Limiting Principles | 14 | amenity, causation, contributory, foreseeability, foreseeable, inconvenience, mitigation, non-monetary, occasioned, remoteness, unforeseeable, vexation, well-being, windfall |
| Restitution and Gain-Based Remedies | 10 | disgorge, disgorgement, enrichment, forfeiture, gain-based, recompense, reliance-based, restitution, restitutionary, set-off |
| Group total | 64 | — |

| Group 7: Interpretation and Construction n = 59 · 7.9% of CLKL |
| --- |

| Sub-category | n | Keywords (alphabetical) |
| --- | --- | --- |
| Process and Standards of Construction | 19 | ascertained, ascertaining, bystander, caveat, characterisation, construe, construed, construing, countenance, decision-making, efficacy, exemplified, exposition, materiality, maxim, reconcile, referential, taxonomy, transparencies |
| Intention and Meaning | 17 | contemplate, contemplated, contemplation, evinced, impute, manifested, notional, obscurity, purport, purported, purporting, purports, putative, subjective, subjectively, supposition, unequivocal |
| Approaches and Methods of Interpretation | 14 | all-or-nothing, axiomatic, axioms, case-by-case, commonsense, formalist, judge-made, literalism, manifest, mischief, orthodox, overt, rewrite, rewriting |
| Evidence and Contextual Materials | 9 | extrinsic, gloss, intelligible, matrix, referable, restatement, subsection, treatise, treatises |
| Group total | 59 | — |

| Group 8: Breach and Performance n = 54 · 7.2% of CLKL |
| --- |

| Sub-category | n | Keywords (alphabetical) |
| --- | --- | --- |
| Breach and its Forms | 17 | breach, breached, breaches, breaching, breaker, defaulted, defaulting, defective, defects, desertion, non-compliance, non-conforming, non-performance, non-performing, tort, tortious, trespass |
| Performance and Non-Performance | 15 | counter-performance, dishonoured, eventuate, foregone, forgo, fulfil, honoured, part-performance, readiness, redelivery, render, renders, self-induced, unload, unperformed |
| Repudiation and Anticipatory Breach | 12 | anticipatory, insistence, insisting, non-breaching, renege, repudiate, repudiated, repudiates, repudiating, repudiation, repudiatory, subvert |
| Payment and Financial Obligations | 10 | accrued, arrears, instalment, instalments, non-delivery, non-payment, part-payment, payable, prepayment, suspensory |
| Group total | 54 | — |

| Group 9: Contract Formation n = 52 · 7.0% of CLKL |
| --- |

| Sub-category | n | Keywords (alphabetical) |
| --- | --- | --- |
| Offer and Invitation to Treat | 12 | advert, bid, counter-offer, intimation, proffered, puff, puffs, tendered, tendering, tenders, unaccepted, unsolicited |
| Consideration | 12 | affection, bargained-for, conferment, counter-promise, donative, forbearance, gratuitous, necessaries, peppercorn, pre-existing, promise-based, simultaneity |
| Acceptance | 10 | acceptances, assent, assented, assenting, assents, face-to-face, instantaneous, mirror, nod, uttered |
| Intention and Formalities | 10 | executed, formalities, pre-contract, pre-contractual, promise-keeping, seal, unsigned, voluntariness, vow, vows |
| Negotiation and Communication | 8 | negotiate, negotiated, negotiating, negotiation, negotiations, transaction, utterance, utterances |
| Group total | 52 | — |

| Group 10: Procedural and Judicial Language n = 38 · 5.1% of CLKL |
| --- |

| Sub-category | n | Keywords (alphabetical) |
| --- | --- | --- |
| Litigation Process | 15 | appeal, conceded, criticised, criticisms, dissented, extra-judicially, impugned, obiter, overruled, sue, sued, sues, suffice, suffices, suing |
| Pleading and Claims | 14 | adduce, admissible, committal, counter-claim, counterclaimed, demurrer, evidentiary, joinder, plea, plead, pleaded, pleading, proposition, propositions |
| Proof and Evidence | 9 | disprove, factum, irrebuttable, onus, presumptively, rebut, rebuttable, rebutted, rebutting |
| Group total | 38 | — |

| Group 11: Discharge and Termination n = 37 · 5.0% of CLKL |
| --- |

| Sub-category | n | Keywords (alphabetical) |
| --- | --- | --- |
| Discharge by Agreement and Waiver | 11 | affirm, affirmation, discharge, discharged, renegotiate, renegotiated, renegotiation, revocable, revoke, waive, waived |
| Frustration and Supervening Events | 9 | frustrate, frustrated, frustrating, frustration, impracticability, non-occurrence, obviates, supervening, unavailability |
| Rescission | 9 | rescind, rescinded, rescinding, rescinds, rescission, resile, retracted, retraction, undo |
| Termination and Cancellation | 8 | cancel, cancellation, cancelled, cancelling, extinguish, lapse, renunciation, withdraw |
| Group total | 37 | — |

| Group 12: Equity and Legal Doctrines n = 24 · 3.2% of CLKL |
| --- |

| Sub-category | n | Keywords (alphabetical) |
| --- | --- | --- |
| Equity and Equitable Doctrines | 9 | doctrinal, doctrine, doctrines, equitable, equities, equity, fiduciary, fusion, sanctity |
| Estoppel | 7 | acquiesced, acquiescence, estopped, estoppel, estoppels, promissory, proprietary |
| Privity and Contractual Obligations | 4 | mutuality, novation, privity, privy |
| Detrimental Reliance and Bailment | 4 | bailment, detriment, detrimental, detrimentally |
| Group total | 24 | — |

| Group 13: Archaic and Latin Expressions n = 21 · 2.8% of CLKL |
| --- |

| Sub-category | n | Keywords (alphabetical) |
| --- | --- | --- |
| Doctrinal Latin Maxims | 14 | (ab) initio, (contra) proferentem, (ex) gratia, (in) lieu, (inter) praesentes, (jus) quaesitum tertio, (prima) facie, (quantum) meruit, (res) extincta, assumpsit, culpa in contrahendo, emptor, pactum, terrorem |
| Judicial and Evidential Terms | 7 | (per) annum, Hypothesi, delicto, dicta, dictum, proferens, vis-a-vis |
| Group total | 21 | — |
