# Supplementary material for: Developing a contracts law keyword list (CLKL) for academic legal education: A corpus-based, keyness-informed study
Source: PLoS One. 2026 Jul 6;21(7):e0352766. doi: 10.1371/journal.pone.0352766 (PMC13336195; doi:10.1371/journal.pone.0352766)
Supplement: Attachment — Submitted filename: Reviewer Comments.pdf [file pone.0352766.s003.pdf]

Reviewer Comments

This paper examines a pedagogically relevant topic and offers a well-organised, corpus-based attempt to construct a CLKL from academic textbooks. It holds potential significance for ELP and legal vocabulary pedagogy, particularly in enhancing students' understanding and usage of legal terminology in academic contexts. However, this paper needs further revisions before it can be published.

Firstly, your work has to be clearer about what it adds to the field of study (***aka. scholarly contribution***). At this time, the key contribution appears to be the creation of another specialised word list, this time based on texts on contract law instead of contracts. This is useful, but the paper should make it clearer what contributions (i.e. ***new methodological*** or ***conceptual contributions***) it makes beyond just building on earlier work or extending the existing literature.

Secondly, the ***Results and Discussion section (pages 21-35)*** is so long and has some repetitive parts. The tier-by-tier presentation repeats similar observations, particularly regarding the predominance of nouns and the relative distribution of adjectives and verbs. This makes your paper's analysis less powerful. I suggest the author shorten these parts and interpret the major findings more synthetically. ***As long as you present the discussion more clearly, you don't need to separate the Results and Discussion sections.***

Thirdly, the pedagogical implications ***(mainly appearing in possible classroom intervention using the CLKL*** section: Page 33) are reasonable but remain rather general.) are reasonable but remain rather general. ***The discussion would be stronger if the author linked specific properties of the CLKL more directly to specific classroom applications.*** At present, the teaching suggestions read more like general EAP/ESP recommendations than implications emerging directly from the present dataset.

Fourthly, the data availability information appears inconsistent and should be checked carefully. In the submission materials (Page 5 in submitted version), the manuscript indicates that ***restrictions apply***, but it also states that ***ALL relevant data are within the manuscript and supporting files*** (Page 6 in submitted version). This discrepancy should be clarified to ensure full consistency with journal policy.

**Data Availability**<br><br>Authors are required to make all data underlying the findings described fully available, without restriction, and from the time of publication. PLOS allows rare exceptions to address legal and ethical concerns. See the PLOS Data Policy and FAQ for detailed information.	No - some restrictions will apply

Powered by Editorial Manager® and ProduXion Manager® from Aries Systems Corporation

**Describe where the data may be found in full sentences. If you are copying our sample text, replace any instances of XXX with the appropriate details.**	All relevant data are within the manuscript and its supporting information files

There are also a few smaller issues. Some novelty claims should be moderated, given that the manuscript itself cites prior contract-law-related vocabulary studies. In addition, one self-citation is still incomplete and marked "***journal details to be added*** (**Page 38**)," which should be corrected.

Overall, this paper is potentially valuable, but in its current version it is more descriptive than analytically strong. I therefore recommend ***major revision***.
