# Supplementary material for: Developing a contracts law keyword list (CLKL) for academic legal education: A corpus-based, keyness-informed study
Source: PLoS One. 2026 Jul 6;21(7):e0352766. doi: 10.1371/journal.pone.0352766 (PMC13336195; doi:10.1371/journal.pone.0352766)
Supplement: Peer review history

# Peer review: Decision Letter 0

**DOI:** 10.1371/journal.pone.0352766.r001

**Authors:** Rachel Chan

14 Apr 2026

PONE-D-26-06991

Developing a contracts law keyword list (CLKL) for academic legal education: A corpus-based, keyness-informed study

PLOS One

Dear Dr. Alasmary,

Thank you for submitting your manuscript to PLOS ONE. After careful consideration, we feel that it has merit but does not fully meet PLOS ONE’s publication criteria as it currently stands. Therefore, we invite you to submit a revised version of the manuscript that addresses the points raised during the review process.

Dear Author

The reviewers have recommended a Major Revision.

The comments are below.

Best regards.

Please submit your revised manuscript by May 29 2026 11:59PM. If you will need more time than this to complete your revisions, please reply to this message or contact the journal office at plosone@plos.org. When you’re ready to submit your revision, log on to https://www.editorialmanager.com/pone/ and select the 'Submissions Needing Revision' folder to locate your manuscript file.

Please include the following items when submitting your revised manuscript:

- A letter that responds to each point raised by the academic editor and reviewer(s). You should upload this letter as a separate file labeled 'Response to Reviewers'.
- A marked-up copy of your manuscript that highlights changes made to the original version. You should upload this as a separate file labeled 'Revised Manuscript with Track Changes'.
- An unmarked version of your revised paper without tracked changes. You should upload this as a separate file labeled 'Manuscript'.

If you would like to make changes to your financial disclosure, please include your updated statement in your cover letter. Guidelines for resubmitting your figure files are available below the reviewer comments at the end of this letter.

If applicable, we recommend that you deposit your laboratory protocols in protocols.io to enhance the reproducibility of your results. Protocols.io assigns your protocol its own identifier (DOI) so that it can be cited independently in the future. For instructions see: https://journals.plos.org/plosone/s/submission-guidelines#loc-laboratory-protocols. Additionally, PLOS ONE offers an option for publishing peer-reviewed Lab Protocol articles, which describe protocols hosted on protocols.io. Read more information on sharing protocols at https://plos.org/protocols?utm_medium=editorial-email&utm_source=authorletters&utm_campaign=protocols.

As the corresponding author, your ORCID iD is verified in the submission system and will appear in the published article. PLOS supports the use of ORCID, and we encourage all coauthors to register for an ORCID iD and use it as well. Please encourage your coauthors to verify their ORCID iD within the submission system before final acceptance, as unverified ORCID iDs will not appear in the published article. *Only*  the individual author can complete the verification step; PLOS staff *cannot*  verify ORCID iDs on behalf of authors.

We look forward to receiving your revised manuscript.

Kind regards,

Rachel Suet Kay Chan, Ph.D.

Academic Editor

PLOS One

Journal Requirements:

When submitting your revision, we need you to address these additional requirements.

1. Please ensure that your manuscript meets PLOS ONE's style requirements, including those for file naming. The PLOS ONE style templates can be found at

https://journals.plos.org/plosone/s/file?id=wjVg/PLOSOne_formatting_sample_main_body.pdf and

https://journals.plos.org/plosone/s/file?id=ba62/PLOSOne_formatting_sample_title_authors_affiliations.pdf

2. Please update your submission to use the PLOS LaTeX template. The template and more information on our requirements for LaTeX submissions can be found at http://journals.plos.org/plosone/s/latex.

3. Thank you for stating the following in the Acknowledgments Section of your manuscript:

“The author gratefully acknowledges the institutional support provided by King Saud University.”

We note that you have provided funding information that is not currently declared in your Funding Statement. However, funding information should not appear in the Acknowledgments section or other areas of your manuscript. We will only publish funding information present in the Funding Statement section of the online submission form.

Please remove any funding-related text from the manuscript and let us know how you would like to update your Funding Statement. Currently, your Funding Statement reads as follows:

“The author extends their appreciation to the deanship of Scientific Research at King Saud University for funding this research”

Please include your amended statements within your cover letter; we will change the online submission form on your behalf.

4. Thank you for stating the following financial disclosure:

“The author extends their appreciation to the deanship of Scientific Research at King Saud University for funding this research”

Please state what role the funders took in the study.  If the funders had no role, please state: "The funders had no role in study design, data collection and analysis, decision to publish, or preparation of the manuscript."

If this statement is not correct you must amend it as needed.

Please include this amended Role of Funder statement in your cover letter; we will change the online submission form on your behalf.

5. Thank you for stating the following financial disclosure:

“The author extends their appreciation to the deanship of Scientific Research at King Saud University for funding this research”

We note that one or more of the authors is affiliated with the funding organization, indicating the funder may have had some role in the design, data collection, analysis or preparation of your manuscript for publication; in other words, the funder played an indirect role through the participation of the co-authors. If the funding organization did not play a role in the study design, data collection and analysis, decision to publish, or preparation of the manuscript and only provided financial support in the form of authors' salaries and/or research materials, please do the following:

1. Review your statements relating to the author contributions, and ensure you have specifically and accurately indicated the role(s) that these authors had in your study. These amendments should be made in the online form.

2. Confirm in your cover letter that you agree with the following statement, and we will change the online submission form on your behalf:

“The funder provided support in the form of salaries for authors [insert relevant initials], but did not have any additional role in the study design, data collection and analysis, decision to publish, or preparation of the manuscript. The specific roles of these authors are articulated in the ‘author contributions’ section.

6. Please upload a new copy of Figure 10 as the detail is not clear. Please follow the link for more information:  https://journals.plos.org/plosone/s/figures

7. If the reviewer comments include a recommendation to cite specific previously published works, please review and evaluate these publications to determine whether they are relevant and should be cited. There is no requirement to cite these works unless the editor has indicated otherwise.

[Note: HTML markup is below. Please do not edit.]

Reviewers' comments:

Reviewer's Responses to Questions

**Comments to the Author**

1. Is the manuscript technically sound, and do the data support the conclusions?

The manuscript must describe a technically sound piece of scientific research with data that supports the conclusions. Experiments must have been conducted rigorously, with appropriate controls, replication, and sample sizes. The conclusions must be drawn appropriately based on the data presented.

Reviewer #1: Yes

Reviewer #2: Yes

**********

2. Has the statistical analysis been performed appropriately and rigorously?

Reviewer #1: N/A

Reviewer #2: Yes

**********

3. Have the authors made all data underlying the findings in their manuscript fully available?

The PLOS Data policy requires authors to make all data underlying the findings described in their manuscript fully available without restriction, with rare exception (please refer to the Data Availability Statement in the manuscript PDF file). The data should be provided as part of the manuscript or its supporting information, or deposited to a public repository. For example, in addition to summary statistics, the data points behind means, medians and variance measures should be available. If there are restrictions on publicly sharing data—e.g. participant privacy or use of data from a third party—those must be specified.

Reviewer #1: No

Reviewer #2: Yes

**********

4. Is the manuscript presented in an intelligible fashion and written in standard English?

PLOS ONE does not copyedit accepted manuscripts, so the language in submitted articles must be clear, correct, and unambiguous. Any typographical or grammatical errors should be corrected at revision, so please note any specific errors here.

Reviewer #1: Yes

Reviewer #2: Yes

**********

5. Review Comments to the Author

Please use the space provided to explain your answers to the questions above. You may also include additional comments for the author, including concerns about dual publication, research ethics, or publication ethics. (Please upload your review as an attachment if it exceeds 20,000 characters)

Reviewer #1: I do not have comment. This paper is ready to be published.

Reviewer #2: Reviewer Comments

This paper examines a pedagogically relevant topic and offers a well-organised, corpus-based attempt to construct a CLKL from academic textbooks. It holds potential significance for ELP and legal vocabulary pedagogy, particularly in enhancing students' understanding and usage of legal terminology in academic contexts. However, this paper needs further revisions before it can be published.

Firstly, your work has to be clearer about what it adds to the field of study (aka. scholarly contribution). At this time, the key contribution appears to be the creation of another specialised word list, this time based on texts on contract law instead of contracts. This is useful, but the paper should make it clearer what contributions (i.e., new methodological or conceptual contributions) it makes beyond just building on earlier work or extending the existing literature.

Secondly, the Results and Discussion section (pages 21-35) is so long and has some repetitive parts. The tier-by-tier presentation repeats similar observations, particularly regarding the predominance of nouns and the relative distribution of adjectives and verbs. This approach makes your paper’s analysis less powerful. I suggest the author shorten these parts and interpret the major findings more synthetically. As long as you present the discussion more clearly, you don’t need to separate the Results and Discussion sections.

Thirdly, the pedagogical implications (mainly appearing in possible classroom intervention using the CLKL section: Page 33) are reasonable but remain rather general) are reasonable but remain rather general. The discussion would be stronger if the author linked specific properties of the CLKL more directly to specific classroom applications. At present, the teaching suggestions read more like general EAP/ESP recommendations than implications emerging directly from the present dataset.

Fourthly, the data availability information appears inconsistent and should be checked carefully. In the submission materials (Page 5 in submitted version), the manuscript indicates that restrictions apply, but it also states that ALL relevant data are within the manuscript and supporting files (Page 6 in submitted version). This discrepancy should be clarified to ensure full consistency with journal policy.

There are also a few smaller issues. Some novelty claims should be moderated, given that the manuscript itself cites prior contract-law-related vocabulary studies. In addition, one self-citation is still incomplete and marked “journal details to be added (Page 38),” which should be corrected.

Overall, this paper is potentially valuable, but in its current version it is more descriptive than analytically strong. I therefore recommend major revision.

**********

6. PLOS authors have the option to publish the peer review history of their article (what does this mean?). If published, this will include your full peer review and any attached files.

If you choose “no”, your identity will remain anonymous but your review may still be made public.

**Do you want your identity to be public for this peer review?**  For information about this choice, including consent withdrawal, please see our Privacy Policy.

Reviewer #1: No

Reviewer #2: No

**********

[NOTE: If reviewer comments were submitted as an attachment file, they will be attached to this email and accessible via the submission site. Please log into your account, locate the manuscript record, and check for the action link "View Attachments". If this link does not appear, there are no attachment files.]

To ensure your figures meet our technical requirements, please review our figure guidelines: https://journals.plos.org/plosone/s/figures

You may also use PLOS’s free figure tool, NAAS, to help you prepare publication quality figures: https://journals.plos.org/plosone/s/figures#loc-tools-for-figure-preparation.

NAAS will assess whether your figures meet our technical requirements by comparing each figure against our figure specifications.

Attachment Submitted filename: Reviewer Comments.pdf

# Author comment: Author response to Decision Letter 1

**DOI:** 10.1371/journal.pone.0352766.r002

29 May 2026

Response to reviewers

I am grateful for the thoughtful feedback provided by the reviewers. All comments raised by the reviewers have been carefully addressed. Here is a point-by-point account of the author's response.

1. Please ensure that your manuscript meets PLOS ONE's style requirements, including those for file naming.

R1. The revised manuscript is reformatted to meet the publication requirement of PLOS ONE.

2. Thank you for stating the following in the Acknowledgments Section of your manuscript:

“The author gratefully acknowledges the institutional support provided by King Saud University.”

We note that you have provided funding information that is not currently declared in your Funding Statement. However, funding information should not appear in the Acknowledgments section or other areas of your manuscript. We will only publish funding information present in the Funding Statement section of the online submission form.

Please remove any funding-related text from the manuscript and let us know how you would like to update your Funding Statement. Currently, your Funding Statement reads as follows:

“The author extends their appreciation to the deanship of Scientific Research at King Saud University for funding this research”

Please include your amended statements within your cover letter; we will change the online submission form on your behalf.

R2. All funding information has been removed from the manuscript. An amended statement is included in the cover letter which shows the number of the funding project which has been obtained recently.

3. Please upload a new copy of Figure 10 as the detail is not clear. Please follow the link for more information: https://journals.plos.org/plosone/s/figures

R3. All figures have been updated and revised to mirror changes in the body of the manuscript as recommended by the reviewers in comment No. 6 below.

4. First, the paper needs to articulate its scholarly contribution more clearly. At present, the principal contribution appears to be the creation of another specialized word list, this time derived from contract law textbooks rather than contractual documents. While this is undoubtedly useful, the manuscript should clarify what methodological or conceptual advances it offers beyond extending previous work or applying established procedures to a new dataset.

R4. This section has been rewritten to demonstrate the full range of contributions of this study.

5. Second, the Results and Discussion section (pp. 21–35) is overly long and contains considerable repetition. The tier-by-tier presentation frequently reiterates similar observations, particularly regarding the predominance of nouns and the relative distribution of adjectives and verbs. As a result, the analysis loses some of its interpretive force. The discussion would benefit from a more synthetic treatment of the findings, with greater emphasis on overarching patterns and their implications. Provided that the analysis is presented more cohesively, there may be no need to maintain separate Results and Discussion sections.

R5. This section has been entirely rewritten to address the concerns raised by the reviewer. In this revised submission, a thematically driven approach is pursued rather than a tier-by-tier presentation. Patterns emerging form the analysis are discussed considering examples from the data. for example, a great deal of keywords is found to denote actors, agents, and parties involved in contractual agreements.

6. Thirdly, the pedagogical implications (mainly appearing in possible classroom intervention using the CLKL section: Page 33) are reasonable but remain rather general.) are reasonable but remain rather general. The discussion would be stronger if the author linked specific properties of the CLKL more directly to specific classroom applications. At present, the teaching suggestions read more like general EAP/ESP recommendations than implications emerging directly from the present dataset.

R6. The purpose of this section is to alert EAP/ESP instructors to the evidence-based, research-informed approaches to second language vocabulary instruction. These approaches are valid no matter what the target vocabulary item may be. For example, there seems to be a consensus among researchers that contextualizing words is conceived to better learning than giving them as separate, isolated instances of the discourse. A short paragraph has been added to this section for the purpose of sequencing topics in a way comprehensible to the students.

7. Fourthly, the data availability information appears inconsistent and should be checked carefully. In the submission materials (Page 5 in submitted version), the manuscript indicates that restrictions apply, but it also states that ALL relevant data are within the manuscript and supporting files (Page 6 in submitted version). This discrepancy should be clarified to ensure full consistency with journal policy.

R7. Data availability statement has been included.

8. There are also a few smaller issues. Some novelty claims should be moderated, given that the manuscript itself cites prior contract-law-related vocabulary studies. In addition, one self citation is still incomplete and marked “journal details to be added (Page 38),” which should be corrected.

R9.

R8. The manuscript has been thoroughly revised to soften the stance on the novelty of the items. However, the comprehensive nature of the list and the methodology used to elicit items on it are genuine contributions of this paper. All journal details have been added to the list of references.

# Peer review: Decision Letter 1

**DOI:** 10.1371/journal.pone.0352766.r003

**Authors:** Rachel Chan, Rachel Chan

15 Jun 2026

Developing a contracts law keyword list (CLKL) for academic legal education: A corpus-based, keyness-informed study

PONE-D-26-06991R1

Dear Dr. Alasmary,

We’re pleased to inform you that your manuscript has been judged scientifically suitable for publication and will be formally accepted for publication once it meets all outstanding technical requirements.

Within one week, you’ll receive an e-mail detailing the required amendments. When these have been addressed, you’ll receive a formal acceptance letter and your manuscript will be scheduled for publication.

An invoice will be generated when your article is formally accepted. Please note, if your institution has a publishing partnership with PLOS and your article meets the relevant criteria, all or part of your publication costs will be covered. Please make sure your user information is up-to-date by logging into Editorial Manager at Editorial Manager® and clicking the ‘Update My Information' link at the top of the page. For questions related to billing, please contact billing support.

If your institution or institutions have a press office, please notify them about your upcoming paper to help maximize its impact. If they’ll be preparing press materials, please inform our press team as soon as possible -- no later than 48 hours after receiving the formal acceptance. Your manuscript will remain under strict press embargo until 2 pm Eastern Time on the date of publication. For more information, please contact onepress@plos.org.

Kind regards,

Rachel Suet Kay Chan, Ph.D.

Academic Editor

PLOS One

Additional Editor Comments (optional):

Reviewers' comments:

Reviewer's Responses to Questions

**Comments to the Author**

1. If the authors have adequately addressed your comments raised in a previous round of review and you feel that this manuscript is now acceptable for publication, you may indicate that here to bypass the “Comments to the Author” section, enter your conflict of interest statement in the “Confidential to Editor” section, and submit your "Accept" recommendation.

Reviewer #2: All comments have been addressed

**********

2. Is the manuscript technically sound, and do the data support the conclusions?

The manuscript must describe a technically sound piece of scientific research with data that supports the conclusions. Experiments must have been conducted rigorously, with appropriate controls, replication, and sample sizes. The conclusions must be drawn appropriately based on the data presented.

Reviewer #2: Yes

**********

3. Has the statistical analysis been performed appropriately and rigorously?

Reviewer #2: Yes

**********

4. Have the authors made all data underlying the findings in their manuscript fully available?

The PLOS Data policy requires authors to make all data underlying the findings described in their manuscript fully available without restriction, with rare exception (please refer to the Data Availability Statement in the manuscript PDF file). The data should be provided as part of the manuscript or its supporting information, or deposited to a public repository. For example, in addition to summary statistics, the data points behind means, medians and variance measures should be available. If there are restrictions on publicly sharing data—e.g. participant privacy or use of data from a third party—those must be specified.

Reviewer #2: Yes

**********

5. Is the manuscript presented in an intelligible fashion and written in standard English?

PLOS ONE does not copyedit accepted manuscripts, so the language in submitted articles must be clear, correct, and unambiguous. Any typographical or grammatical errors should be corrected at revision, so please note any specific errors here.

Reviewer #2: Yes

**********

6. Review Comments to the Author

Please use the space provided to explain your answers to the questions above. You may also include additional comments for the author, including concerns about dual publication, research ethics, or publication ethics. (Please upload your review as an attachment if it exceeds 20,000 characters)

Reviewer #2: Thank you for your further revisions and for your valuable contribution to this area. In my view, the manuscript is now suitable for publication in PLOS ONE.

**********

7. PLOS authors have the option to publish the peer review history of their article (what does this mean?). If published, this will include your full peer review and any attached files.

If you choose “no”, your identity will remain anonymous but your review may still be made public.

**Do you want your identity to be public for this peer review?**  For information about this choice, including consent withdrawal, please see our Privacy Policy.

Reviewer #2: **Yes:** Qiang Li

**********

# Editor report: Acceptance letter

**DOI:** 10.1371/journal.pone.0352766.r004

**Authors:** Rachel Chan, Rachel Chan

PONE-D-26-06991R1

PLOS One

Dear Dr. Alasmary,

I'm pleased to inform you that your manuscript has been deemed suitable for publication in PLOS One. Congratulations! Your manuscript is now being handed over to our production team.

At this stage, our production department will prepare your paper for publication. This includes ensuring the following:

* All references, tables, and figures are properly cited

* All relevant supporting information is included in the manuscript submission,

* There are no issues that prevent the paper from being properly typeset

You will receive further instructions from the production team, including instructions on how to review your proof when it is ready. Please keep in mind that we are working through a large volume of accepted articles, so please give us a few days to review your paper and let you know the next and final steps.

Lastly, if your institution or institutions have a press office, please let them know about your upcoming paper now to help maximize its impact. If they'll be preparing press materials, please inform our press team within the next 48 hours. Your manuscript will remain under strict press embargo until 2 pm Eastern Time on the date of publication. For more information, please contact onepress@plos.org.

You will receive an invoice from PLOS for your publication fee after your manuscript has reached the completed accept phase. If you receive an email requesting payment before acceptance or for any other service, this may be a phishing scheme. Learn how to identify phishing emails and protect your accounts at https://explore.plos.org/phishing.

If we can help with anything else, please email us at customercare@plos.org.

Thank you for submitting your work to PLOS ONE and supporting open access.

Kind regards,

PLOS ONE Editorial Office Staff

on behalf of

Dr. Rachel Suet Kay Chan

Academic Editor

PLOS One
